# Symmetries in metabolic networks of *Escherichia coli*

**DOI:** 10.1093/pnasnexus/pgaf080

**Published:** 2025-03-17

**Authors:** Luis Alvarez, Kuang Liu, Cecilia Ishida, Mishael Sánchez-Pérez, Stefan Wuchty, Hernán A Makse

**Affiliations:** Levich Institute and Department of Physics, City College of New York, New York, NY 10031, USA; Levich Institute and Department of Physics, City College of New York, New York, NY 10031, USA; Faculty of Medicine and Biomedical Sciences, Autonomous University of Chihuahua, Chihuahua 31125, Mexico; Centro de Ciencias Genómicas, Universidad Nacional Autónoma de México, Cuernavaca 62210, Mexico; Department of Computer Science, University of Miami, Coral Gables, FL 33146, USA; Department of Biology, University of Miami, Coral Gables, FL 33146, USA; Institute of Data Science and Computing, University of Miami, Coral Gables, FL 33146, USA; Sylvester Comprehensive Cancer Center, University of Miami, Miami, FL 33136, USA; Levich Institute and Department of Physics, City College of New York, New York, NY 10031, USA

**Keywords:** metabolic networks, symmetries, fibrations, Fibonacci structures

## Abstract

The over-representation of motifs was previously considered a viable definition of building blocks in biological networks. Here, we construct an alternative definition based on invariance properties of enzymes in metabolic networks of *Escherichia coli*. In particular, we consider input trees of each enzyme that bundle all metabolic reactions where information is transmitted. Isomorphisms of such input trees point to symmetric enzymes grouped in “fibers” of the metabolic network that process equivalent dynamics. Such groups of enzymes constitute an alternative concept of building blocks which can be systematically classified into topological types of input trees according to their complexity. In contrast to motifs and modules, enzymes in such fibers are not necessarily mutually connected but still can be functionally related. Our analysis finds novel varieties of building blocks that capture such symmetries in hitherto unknown “composite Fibonacci” fibers. Lending credence to their significance as fundamental building blocks, we observe that enzymes in fibers are functionally more homogeneous than their network motif and module counterparts, suggesting that fibers point to a novel way of building blocks that capture metabolic functionality on a topological level.

**Significance Statement**

In contrast to network motifs that were previously heralded as building blocks in biological networks, we introduce an alternative definition that revolves around “fibers” in metabolic networks of *Escherichia coli*. In particular, such fibers constitute groups of “symmetric” enzymes in that their network inputs are equivalent, pointing to overlapping dynamics. Notably, we show their significance as novel building blocks that capture metabolic functionality on a topological level through their heightened functional homogeneity compared to their network motif and module counterparts.

## Introduction

A central tenet of systems biology is the presence of fundamental building blocks that drive the emergence of complex collective behavior (1, 2). Previously, simple network motifs were considered as such building blocks of biological networks (1, 3, 4) that appear in gene regulatory networks (1, 3–5), metabolic networks (6), cellular processes, pathways and ecosystems as well as social and infrastructure networks (7). Despite their significant appearance, the question remains if such network motifs carry a functional role in biological networks (8–10).

Alternatively, the fundamental building blocks of matter in physical systems are uncovered by group symmetries that define the fundamental particles in nature (11). A more general symmetry, called fibration symmetry (12), has been discovered through the analysis of transcriptional regulatory networks (TRN) of *Escherichia coli* and other species (13, 14). These symmetries are less constrained than symmetry groups as they form groupoids (15), defined as groups absent from the composition law. In Refs. (13, 14), the dynamics of gene synchronization was captured by different types of isomorphic input trees that form symmetry fibrations. Specifically, genes that were described by isomorphic input trees were grouped in clusters of genes called “fibers” (13, 14) that point to synchronized building blocks of biological networks. Such graph fibrations (12, 15, 16) generalize the fiber bundles of physics (11) and provide a way to reduce complex networks by capturing important structural and dynamic features. Such transformations preserve the dynamics of information flow in the network, pointing to a coherent explanation of complex patterns of gene coexpression (17).

While the original fibration analysis (13, 14, 17) was limited to the TRN of *E. coli*, we focus on metabolic networks, where enzymes transform metabolites through metabolic reactions. By constructing directed networks of enzymes in *E. coli*, we determine fibers in the corresponding representation of enzyme webs. Specifically, directed links in these networks are akin to the passing of metabolic information messages emanating from the source and ending at target enzymes. Such a view is related to distributed computational networked systems (12), where the behavior of network nodes is fiberwise constant, in that processors in the same fiber are in the same state. Analogously, enzymes in fibers are synchronous in a similar way akin to processing units that are synchronous in a computer. Such an assumption suggests that enzyme-based fibers are elementary building blocks that bundle metabolic information in metabolic pathways, pointing to a novel way of considering metabolic dynamics on a topological level.

Performing a graph fibration analysis of the metabolic network of *E. coli* we uncover novel *fibration building blocks* of much larger complexity than observed before, indicating the ways such networks are built from the bottom up. Compared to the TRN of *E. coli* (14), which is mostly dominated by simple fiber structures where single proteins regulate fibers, we identify more complex structures in metabolic networks where fibers regulate other fibers. Such a complexity is expressed in a richer set of metabolic fibers with intricate cycles of feed-forward and feedback structures that are captured by a systematic classification in terms of fractal dimensions.

To indicate their biological relevance, we find that enzymes in fibers are functionally more homogeneous than enzymes in network motifs (3) and network modules (18). Such observations point to a hitherto unknown level of complexity in the organization and architecture of biological networks, that topological motifs and modules through statistical means simply miss. Furthermore, as fibers represent information flow that goes beyond the boundaries of statistical overrepresentation of interactions between sets of nodes, such groups of enzymes may well be a better entry point not only to elucidate pathways from a different angle but also to find novel pathways. Along the same lines, such fibers may also be used as a way to find new drug targets, as fibers capture information flow, potentially providing an alternative way to indicate points of therapeutic intervention.

## Results

**Fig. 1.**
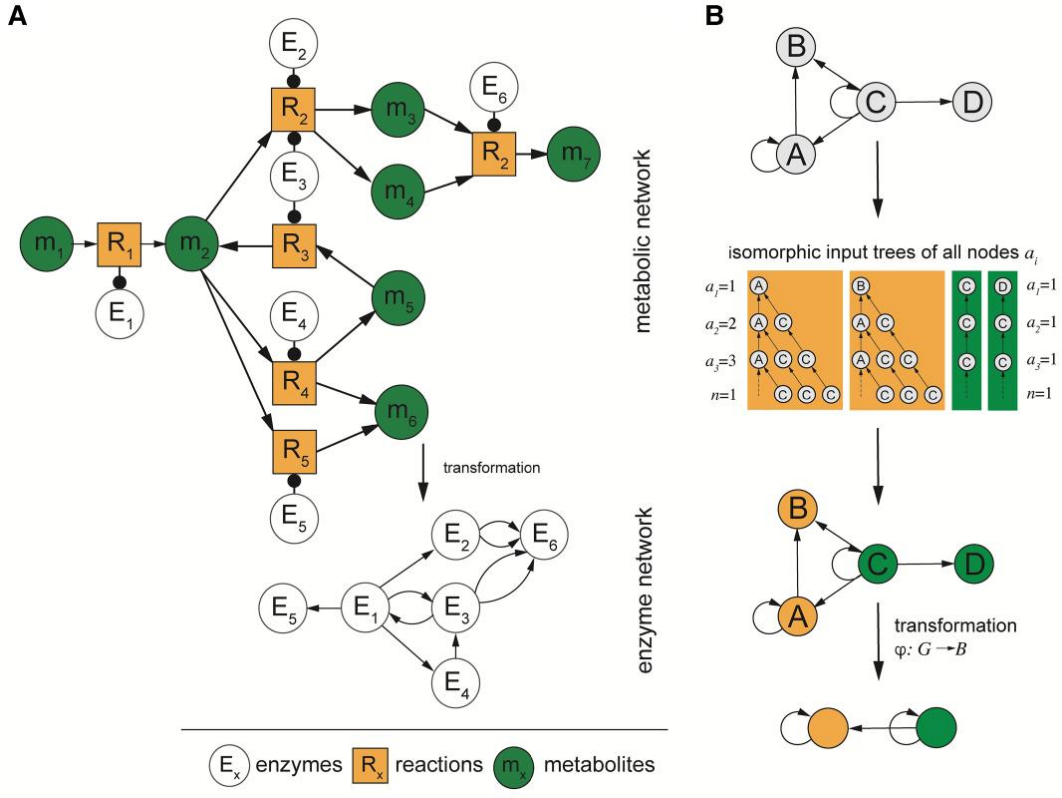
Construction of enzyme metabolic networks and determination of symmetric network characteristics. A) In the toy metabolic model, we connect enzymes (E) through directed links, when the products (*m*) of a preceding reaction (*R*) that is catalyzed by an enzyme are turned into a reactant in the subsequent reaction that is controlled through a different enzyme. B) Using such directed enzyme networks, we determine symmetric characteristics through input trees of each enzyme. Trees at *A* and *B* share isomorphisms through an infinite number of layers as a consequence of the self-loop at *A* and *B*. Similarly, we observe that isomorphic input trees of nodes *C* and *D* are a result of a self-loop at *C*. For each input tree, we show the number of paths of length i−1 at every layer of the input tree, $a_{i}$, and its branching ratio *n*. Such isomorphisms of input trees of *A* and *B* as well as *C* and *D* allow us to transform the whole network *G* into its base *B* by collapsing enzymes in each fiber into one large node through an isomorphic symmetry fibration φ.

Focusing on the latest versions of the Ecocyc (19) and RegulonDB databases (20), we collect metabolic reactions and corresponding enzymes, capturing 2,628 reactions between 2,093 metabolites. After the deletion of ubiquitous metabolites such as ATP or $H_{2}O$, we obtain 2,628 reactions with 1,990 nontrivial metabolites, catalyzed through 1,930 enzymes. To construct an enzyme-specific network (Fig. 1A), we connect enzymes, when the product in a preceding reaction turns into a reactant in the subsequent reaction. As a result, we obtain an enzyme–enzyme network that captures 1,753 enzymes in a web of 22,011 directed edges.

### Fibration formalism

Capturing the flow of information in such a metabolic message-passing network, the information that arrives at a given enzyme contains the entire history that was transmitted through all the paths that end up at the enzyme in question. By classifying enzymes on the basis of their received information, we elucidate the building blocks that are dynamically and functionally important. Specifically, we capture such information through input trees, that provide the full history of information input of a given enzyme.

Formally, in a directed network $G_{e}=(V_{e},E_{e})$ that is composed of $V_{e}$ nodes and $E_{e}$ directed edges (Fig. 1B) the input tree $T_{i}$ of a given node *i* is defined as the tree of all network paths in $G_{e}$ that end at node *i* (14). Specifically, the input set of a node *i* is made up of the series of incoming edges and their respective sources. We consider such an input set of a root node and the corresponding input sets of the root node’s input set in an iterative manner, allowing us to obtain an input tree $T_{i}$ that is rooted at node *i*. In our example in Fig. 1B, we observe that the trees of node *A* and *B* are fed by input from *A* through a self-loop. Similarly, we find that the trees of *C* and *D* are similar since both nodes receive their input from self-loops at node *C*. As a consequence of the construction of input trees, every other node *j* in the underlying tree represents the initial node of a path in the network ending at root *i*. Furthermore, we can represent the structure of the input tree of an enzyme by a sequence, $a_{i}$, that we define as the number of enzymes in the *i*th layer of the input tree, indicating the number of paths of length i−1 that reach the enzyme at the root. Subsequently, the sequence $a_{i}$ can then be characterized by the branching ratio *n* of the input tree defined as


(1) $$\frac{a_{i+1}}{a_{i}}\overset{i\to \infty }{⟶}r.$$

Such a branching ratio indicates the multiplicative growth of the number of paths of length *i* across the network, reaching the enzyme at the root, considered as the fractal dimension of the input tree.

Assuming that metabolites are messengers in a message passing network between enzymes we can draw a connection to distributed computational networked systems (12) that can be broken down by its symmetries, under the assumption that no significant time-delay for any messages exists. In other words, all nodes receive information in the underlying network simultaneously, while their internal computations are homogeneous, suggesting that the input functions of the enzymes are equal. Under these assumptions, we previously found that the structure of transcriptional regulation of genes were much simpler and transparent to understand (21). Applying the same framework to a metabolic network, the results of Boldi and Vigna on fibered distributed computing systems apply (12), indicating that enzymes process the same history, when they are reached by identical paths. As a consequence, their output is equivalent, causing enzymes to synchronize. Such relations are represented by isomorphic input trees in the network that have the same shape but are agnostic of the identity of the underlying nodes. Formally, an isomorphism is defined as a bijective map that preserves the topology of the input trees including the type of links (Fig. 1B). Specifically, a map $\tau \;:\;T\to T^{\prime }$ is an isomorphism if and only if for any pair of connected nodes *a* and *b* of *T*, the pair of nodes τ(a) and τ(b) of T′ are connected by the same type of link.

Nodes that share an isomorphic input tree form a fiber (12, 14), which bundle nodes in the network into nonoverlapping groups. Subsequently, we can construct a building block for each fiber that determines its dynamic behavior, pointing to fibration building blocks. Nodes in the same fiber are synchronous under the previous assumptions, but more generally represent redundant paths of information, given that their paths are multiple copies of each other embedded in the network. Collapsing these redundant information pathways into just a single node does not change the dynamics of the network, given that this procedure does not eliminate any pathways, but instead reduces their redundancies. Such a framework draws a parallel to physics and allows us to understand the set of all input tree isomorphisms as the fibration symmetries of the network (14, 21), pointing to building blocks of the underlying biological network.

Specifically, this process is formalized by the concept of graph fibrations (12), where φ is a morphism


(2) φ:G→B

that maps a graph $G=(N_{G},E_{G})$ to a different graph $B=(N_{B},E_{B})$. Such a construct is labeled the base of the graph fibration φ, preserving the input tree of every node $g\in N_{g}$. This characteristic is more easily understood by the *lifting* property: every edge targeting an image node $\varphi (i)=i^{\prime }$ can be *uniquely* lifted to an edge in *G* targeting *i* (12, 21). Consequently, multiple edges that target the same node in *G* cannot be collapsed to fewer edges in *B* targeting its image node.

In this work, we consider a surjective minimal graph fibration (12), called a symmetry fibration (14), φ, that captures all the symmetries of the network by mapping all nodes to isomorphic input trees to a single node in the base. Each set of collapsed nodes in *G* forms a fiber pointing to a single node in *B* that produces the minimal base of the network, i.e. the maximum compression of *G*. In such a case, base *B* consists of a graph where all enzymes in a fiber are collapsed into one representative node by a symmetry fibration (Fig. 1B). As a consequence, a symmetry fibration leads to a dimensional reduction of the network into its smallest size without disturbing its dynamics. Crucially, symmetry fibration *B* conserves the dynamics of the network such that the reduced network behaves in the same way as the original one:


(3) Dyn(G)=Dyn(B).

As fibers partition the graph *G* into unique and nonoverlapping sets $\Pi =\{\Pi_{1},\ldots ,\Pi_{r}\}$, we denote $i{∼}_{\Pi }j$ when the input trees of *i* and *j* are isomorphic and belong to the same fiber $\Pi_{k}$. Such a concept suggests that $\exists k|i,j\in \Pi_{k}$, and a symmetry fibration exists that sends both nodes to the same node in the base, φ(i)=φ(j) (Fig. 1B). According to these results, we interpret synchronous nodes to process the same information received through isomorphic paths in the network and are interpreted as the building blocks of the system.

To determine fibers in a network we use the “minimal balanced coloring” algorithm (BCA) (14, 15, 22–24) for the computation of minimal bases (25), assuming that the dynamical state of a node is analogous to a process of assigning a color to each enzyme. Each node receives colors from adjacent nodes through incoming links and sends its color to the adjacent nodes through its outgoing links. As a consequence, such color flow is equivalent to input trees, indicating that nodes with the same color profile in the balanced coloring partition (15) correspond to fibers induced by symmetry fibrations (24).

### Fibration analysis of the metabolic network

To consider relevant network components we first find the largest strongly connected component in our network composed of 1,753 enzymes that are embedded in 22,011 directed edges, capturing more than 70% of all enzymes. In turn, we find a few other smaller strongly connected components that are composed of four nodes on average. From the entire enzyme network, we extracted four subnetworks that are related to major metabolism groups. To focus on prominent subnetworks of the entire metabolic network, we initially capture metabolic reactions that revolve around glycolysis as the major bacterial energy resource. Furthermore, we extract carbon metabolic pathways where one-carbon moieties are transferred from donors to intermediate carriers, ultimately used in methylation reactions or in the synthesis of purine and thymidine, which are used in DNA building blocks. As a corollary, we also account for amino acid synthesis pathways as well as oxidative stress as a natural consequence of aerobic metabolism (Table S1).

**Fig. 2.**
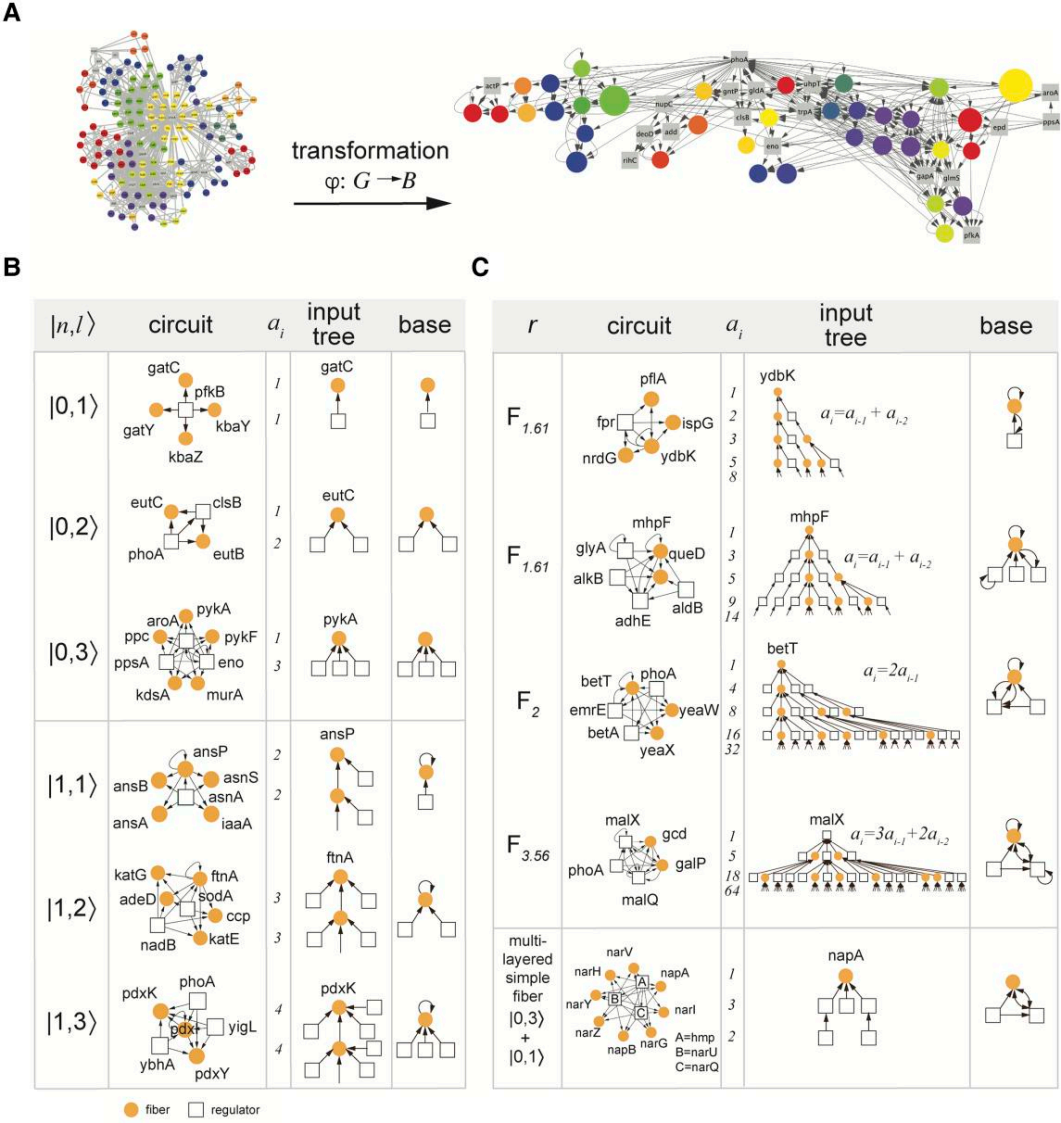
Fibers in the enzyme network. In A), we determine fibers in the glycolysis enzyme network in *E. coli*, where colors refer to fibers. Furthermore, we collapse all enzymes in a fiber into one large node, transforming the whole network *G* into its base *B* through a fiber isomorphic symmetry fibration φ. B) In amino-acid synthesis, carbon metabolism, glycolysis, and oxidative stress enzyme networks, we find basic, annotated fibration building blocks where no information flows back to regulator enzymes that are labeled by |n,l⟩, where *n* is the number of general *n*-ary trees and *l* is the number of regulators. In C), we show examples of complex Fibonacci building blocks, characterized by an auto-regulated fiber that sends information back to its regulator enzymes. Such a characteristic creates a fractal input tree that encodes a Fibonacci sequence with a branching ratio of the number of paths $a_{i}$. Furthermore, we show a multilayer simple fiber in the bottom panel, that combines two feed-forward structures of type |n,l⟩.

Utilizing our BCA algorithm, we find 44 fibers in the amino-acid synthesis network, 32 fibers in the carbon metabolism network, 35 fibers in the glycolysis network (Fig. 2A), and 30 fibers in the oxidative stress network, respectively (Fig. S1). The lists of all fibers of each network can be found in the Supplementary material. The symmetry fibrations φ in these subnetworks allow us to shrink the underlying networks dramatically (Table S2) into a small number of bases (Figs. 2A and S1). Such fibers cover the underlying networks significantly, as the majority of nodes appear in fibers (Table 1). Moreover, at least roughly one half of all the nodes belong to a strongly connected component (SCC) in the networks, while a large fraction of such nodes appear in fibers as well.

**Table 1.** Structural composition of the four enzyme networks.

	Amino acid synthesis		Carbon metabolism		Glycolysis		Oxidative stress	
	Nodes	%	Nodes	%	Nodes	%	Nodes	%
Total nodes	261	100	149	100	153	100	168	100
Nodes in fibers	198	75.9	95	63.8	135	88.2	137	81.5
In SCCs (in fibers)	147 (107)	56.3 (41)	93 (65)	62.4 (43.6)	80 (66)	52.3 (43.1)	80 (62)	47.6 (36.9)
Connectors (in fibers)	18 (5)	6.9 (1.9)	22 (4)	14.8 (2.7)	1 (0)	0.7 (0)	9 (3)	5.4 (1.8)
$k_{\mathrm{out}}$ shell (in fibers)	96 (86)	36.8 (33)	34 (26)	22.8 (17.4)	72 (69)	47.1 (45.1)	79 (72)	47 (42.9)

We show the coverage of each network by fibers as well as the structural breakdown of the network. Strongly connected components (SCC), correspond to interconnected components of the network where each node is accessible to every other node. Typically, the networks consist of four SCCs where one component captures the majority of nodes. Other than belonging to a SCC, enzymes are classified as connector nodes or nodes in the $k_{\mathrm{out}}$ shell. Connector nodes send outputs to the SCCs, while the nodes in the $k_{\mathrm{out}}$ shell only receive information from nodes in the SCCs.

### Fibration building blocks

In the context of our four distinct metabolic networks, we observe a diverse array of input tree topologies (Fig. 2B). While these input trees display considerable variation, they also exhibit common topological features, enabling us to classify all fibration building blocks into fundamental categories (14). Specifically, we construct a building block for a given fiber by evaluating the external incoming nodes (i.e. regulator enzymes) in the corresponding fiber’s input set.

Formally, we define a *fibration building block* as an induced subgraph i.e. defined as a subgraph of a network that includes all edges between the nodes in the subgraph formed by the nodes in the fiber and regulator nodes that send inputs to the fiber. In the case where the fibers send information back to the regulators through a feedback loop from the fiber to the regulators, all nodes that belong to the different shortest paths from the nodes in the fiber to the different regulators are also included in the induced subgraph.

The majority of encountered fibers in previous studies (14, 21) exhibit simple input tree topologies that can be classified using integer values denoted as “fiber numbers” |n,l⟩. Specifically, infinite *n*-ary trees with an integer branching ratio of r=n represent cycles of length *n* intertwined within the core of the fiber. In turn, parameter *l* signifies *l* external regulators, governing the behavior of the fiber.

In particular, most fibers previously observed in *E. coli*’s transcriptional network are of type n=0,1. *n* characterizes the length of the cycles involved in the fiber’s input tree. Furthermore, a value of n=0 corresponds to the absence of cycles between nodes in a fiber. Such an observation points to a finite input tree, indicating a strict feed-forward regulation from the *l* regulators to the nodes in the fiber. While n=1 represents a cycle between the nodes in the fibers, their input trees are infinite trees. However, these input trees show no branches but rather exhibit a chain-like structure. Yet, this classification does not consider the nature of the type of connections within these basic building blocks, which in turn results in additional subcategories of fibers with specific synchronization patterns (Fig. 2B).

While present in the four enzyme networks, this type of simple fibers rather indicate a minority as most of the observed fiber structures correspond to much more complex structures. In particular, the fact that a pair of enzymes can “communicate” through numerous different metabolites means that multiple different edges between the enzyme nodes exist. Such links lead to a more densely connected network structure that produces a high number of cycles between nodes. However, these cycles do not only appear between nodes in the same fiber but between nodes in different fibers. Specifically, we find that such a feature is ubiquitous in metabolic networks and produces much more complex structures than in the transcriptional network of *E. coli* (14, 21).

#### Fibonacci building blocks

In contrast to building blocks that are characterized by an integer branching ratio n=1,2,…, we also find fibers with branching ratios that correspond to a noninteger number *r*. Such circuits contain additional cycles in the building blocks that transform input trees into fractal trees characterized by noninteger fractal branching ratios (Fig. 2C). Turning an input tree into a fractal tree needs (21) (i) a cycle between the fiber and at least one of it’s regulators, ensuring that the fiber nodes send feedback to the regulators, as well as (ii) a self-loop on any of the nodes in the corresponding cycle. Meeting such criteria guarantees that the input tree of the building block branches out with a noninteger branching ratio *r*, obtained by the same limit in Eq. (1), as indicated in Fig. 2C.

Fiber input trees that satisfy both of the mentioned conditions are characterized by recurring relations in the sequence $a_{i}$. Therefore, these structures are called *Fibonacci* Fibers, since the simplest instance of this case precisely follows Fibonacci’s recurrence relation $a_{i}=a_{i-1}+a_{i-2}$. For example, the fiber *pflA-ispG-ydbK-nrdG* in Fig. 2C is represented by a base that features a simple cycle with regulator *fpr*, where the number of paths of length i−1 is characterized by the series 1,2,3,5,8,…, which follows the Fibonacci recurring relation $a_{i}=a_{i-1}+a_{i-2}$ for i>2. Such a sequence of numbers leads to the noninteger branching ratio known as the golden ratio, $r=\frac{a_{i+1}}{a_{i}}\overset{i\to \infty }{⟶}\phi =1.618$. This topology is based on the combination of an auto-regulation loop of the focal node *ydbK* that contributes to the input tree through an infinite chain with branching ratio n=1 and is captured by the first term in the Fibonacci series $a_{i}=a_{i-1}$. A second feedback cycle between *ydbK* and *fpr* sends the information from the fiber to the regulator and back to the fiber, a short-term “memory” effect captured by the second term $a_{i}=a_{i-2}$.

To determine the branching ratio of any input tree *r*, we first need to determine the sequence $a_{i}$ that is the number of nodes in the *i*th layer of the input tree. For small and simple Fibonacci building blocks, the recurrence relation that defines the sequence $a_{i}$ (e.g. $a_{i}=a_{i-1}+a_{i-2}$ for i>2) can be easily determined manually by constructing the input tree for the building block. However, when the recurrence relations point to higher branching ratios, we apply an algorithm, explained in detail in the Methods section. Briefly, we consider the adjacency matrix of the building block and the root node. We iteratively calculate the number of times each node is present in the *i*th generation of the input tree by constructing the input tree step-by-step based on the input relations from the adjacency matrix and the number of times each node appears in the previous generation i−1. $a_{i}$ is then calculated by adding the number of nodes present in that generation. Finally, *r* is calculated by taking the fraction between the last entry in $a_{i}$ and its previous entry. This process is repeated until the value of *r* does not change in consecutive iterations.

#### Multi-Layer simple building blocks

Building blocks can also be combined to produce composite fibration building blocks, helping to systematically understand higher-order functions of biological systems that are composed of many elements (bottom panel, Fig. 2C). We discover a particular type of composite circuit of high complexity that is only found in the metabolic network. In particular, such a fibration building block structure is composed of two elementary fibers, that we label Multi-Layer fibers. The key feature of such a structure is the “composition” of fibers where a fiber is regulated by another fiber in a feed-forward manner.

For instance, the fiber *napA-napB-narG-narH-narI-narV-narY-narZ* in Fig. 2C is regulated by *hmp* as well as the fiber *narU-narQ*, forming a Multi-Layer fiber of type |0,3⟩ that covers the regulation of *napA-napB-narG-narH-narI-narV-narY-narZ*. Furthermore, we also find a |0,1⟩ fiber, that describes the dependence between the three regulators *hmp, narU*, and *narQ*, resulting in a building block that is described as the composition |0,3⟩⊕|0,1⟩. The observed composition through the formal definition of a fibration building block can be considered in the following way: when constructing the building block of the “downstream” regulated fiber we need to account for its regulators, thereby including the regulating fibers and “stitching” the two fibers and their building blocks together.

### Composite Fibonacci fibration building blocks

So far, most of the observed fibration building blocks are of type |n,l⟩ as well as a few Fibonacci and Multi-Layer building blocks (14, 21). All of these building block classes correspond to single fibration building blocks, with the exception of the Multi-Layer simple building blocks, which correspond essentially to a composition of building block structures in a “*sequential manner*,” where one fiber regulates another one.

**Fig. 3.**
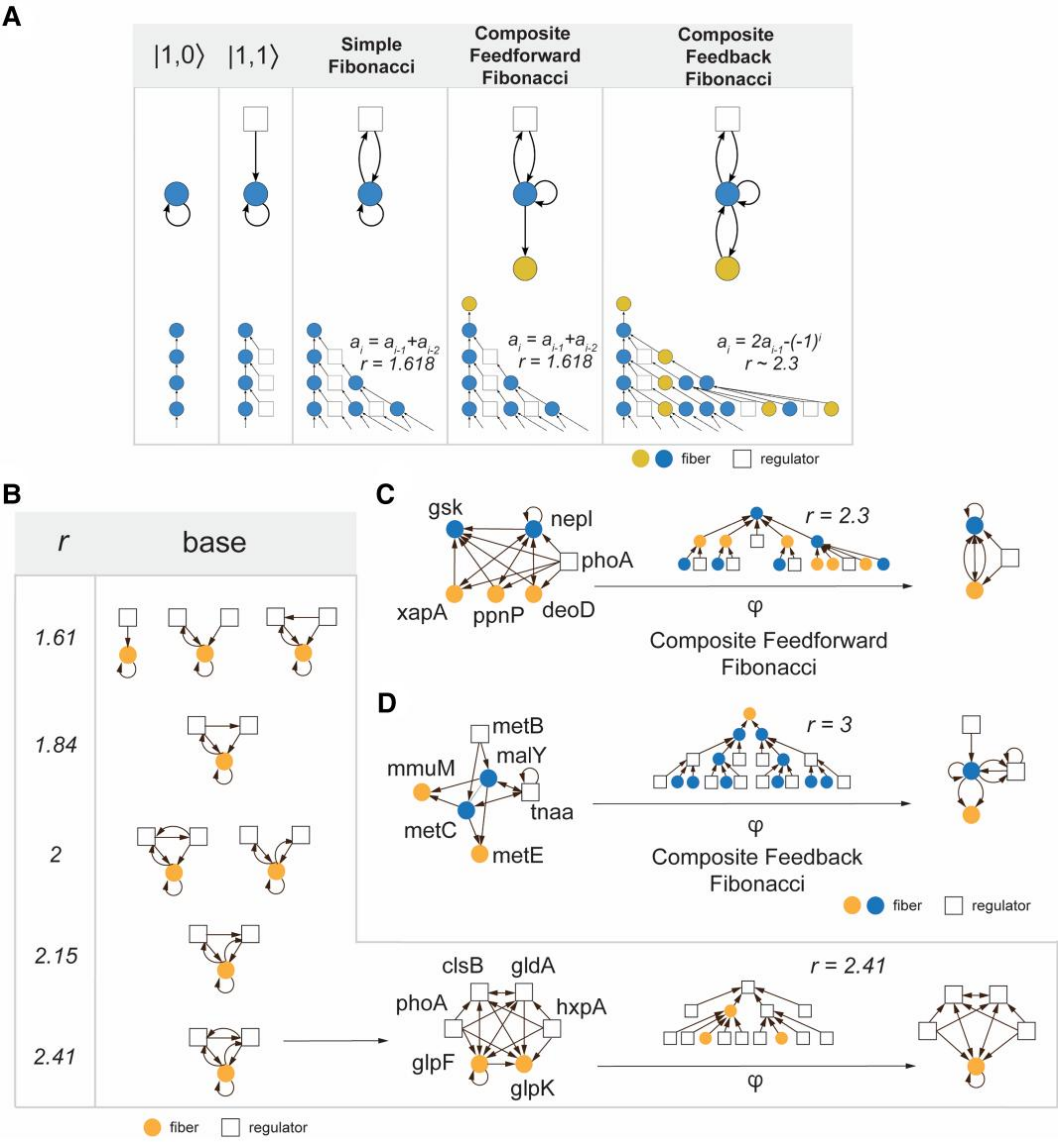
Complexity axes of Fibonacci fibers. A) In a “horizontal axis,” we show the transition from simple to complex types of building blocks through the addition of feedback loops. In particular, we observe that adding a feedback loop between a regulator and a fiber that points to a simple Fibonacci fiber allows the corresponding input tree to branch out. When we add an output edge regulating another fiber we arrive at a *Composite Feedforward Fibonacci*. The addition of a feedback loop to a newly added fiber allows us to create a *Composite Feedback Fibonacci* building block that further branches out. B) A “vertical axis of complexity” corresponds to an increase in complexity through an elevated branching ratio. In particular, such structures emerge with the increased addition of feedback loops between the underlying nodes. We observe such a characteristic in a Composite Feedback Fibonacci fiber revolving around the regulation of *glpFK* through regulators that are connected through mutual feedback loops. C) In a Feedforward Fibonacci, we find a fiber *mmuM-metE* with a branching ratio even though it does not send any feedback to its regulators, as it is in fact regulated by another fiber *metC-malY*. As the blue regulator fiber is part of a Fibonacci structure the input tree of fiber *mmuM-metE* includes the input tree for *malY-metC* which is a branching tree, making its own input tree branch out. D) In a Feedback Fibonacci, two fibers (i.e. blue and yellow nodes) regulate each other, pointing to a branching input tree structure with r=2.3. This type of Fibonacci structure features two (or even more) fibers representing a step further in the complexity of previously observed fibers.

One of the key findings in these enzyme networks is the presence of *Composite Fibonacci* structures (Fig. 3A, C, D), building blocks that are composed of more than one fiber. These composite Fibonacci structures are labeled *Composite Feedforward Fibonaccis* and *Composite Feedback Fibonaccis*. Specifically, building blocks can be composed either through a fiber that regulates another one in a feed-forward manner or by forming a feedback loop between the underlying fibers. In more detail, *Composite FeedForward Fibonaccis* are an instance of a Multi-Layer Fiber, in which a fiber regulates another one in a feed-forward manner, where the regulating fiber belongs to a Fibonacci building block. In particular, the regulated fiber obtains a branching input tree from a regulating Fibonacci fiber, hence allowing its own input tree to branch out. In *Composite Feedback Fibonaccis*, a layer of complexity is added by forming a loop between the fibers such that each fiber acts as a regulator to each other.

To understand the abundance and systematically characterize the complexity of the building blocks we consider two “*complexity axes*,” facilitating the structured organization of building blocks according to their complexity levels. A “horizontal axis” relates the simple to more complex types of building blocks (Fig. 3A). Traversing different bases, we start with the base of |n=1,l=0⟩ that depicts a single fiber regulating itself. The addition of an external regulator allows us to obtain the structure |n=1,l=1⟩. Although both of these input trees are infinite as a result of self-regulation, they do not branch out (Fig. 3A). However, when we add an edge from the fiber back to the regulator, we obtain the simplest Fibonacci fiber with a branching input tree corresponding to r=1.618.

Furthermore, when we add an output edge regulating another fiber we arrive at a *Composite Feedforward Fibonacci*, since the Fibonacci fiber is now acting as a regulator of another fiber downstream. We show this case in Fig. 3A, where we add a yellow fiber that is regulated by a simple Fibonacci Fiber in blue, allowing us to create a Composite Feedforward Fibonacci fiber. The yellow fiber’s input tree is given by the blue fiber’s input tree with the addition of the new yellow node that connects to the root of the input tree of the blue fiber. Since the only difference between these input trees is the newly added yellow node, the branching ratio remains unchanged when we compare the simple Fibonacci and Composite Feedforward Fibonacci fiber. In turn, the addition of yet another edge that connects the new fiber with the original Fibonacci fiber creates a feedback loop, leading to a *Composite Feedback Fiber*. As a consequence, the underlying input tree changes, pointing to a branching ratio of 2.

A second “vertical axis of complexity” corresponds to an increase in complexity through an increasing branching ratio. In Fig. 3B, we increase the branching ratio from the simplest Fibonacci (r=1.618) to a building block with a branching ratio of r=2.41 in a Fibonacci building block that revolves around the regulation of *glpFK* through regulators that are connected through mutual feedback loops (Fig. 3B).

**Fig. 4.**
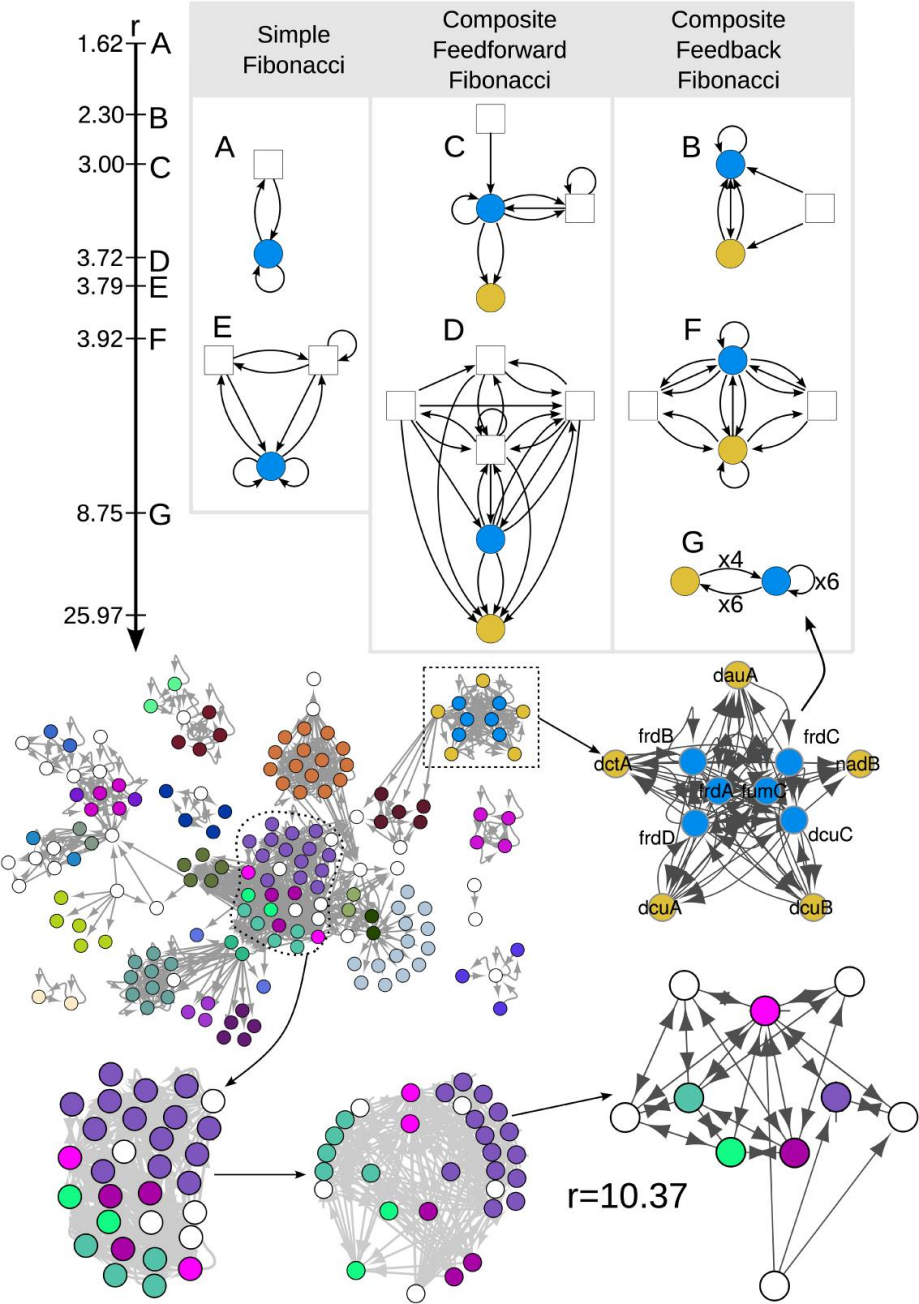
Complexity ladder of the branching ratio *r*. We show the base of different building blocks corresponding to different types of *Fibonaccis*. The axis on the left shows the range of the branching ratios observed in the four different enzyme networks while the indicated labels correspond to the shown building blocks in the table. The first column depicts *Simple* Fibonaccis (i.e. Fibonacci fibers with only one fiber in the building block). The middle column shows three different *Feedback* Fibonaccis that we define as building blocks where the feedback loop between a fiber and its regulator(s) crosses one, or multiple, other fiber(s). The right column depicts *Feedforward* Fibonaccis, where the fiber in a Fibonacci structure (blue fiber) regulates a second fiber (yellow fiber) in a feed-forward manner where no cycles between the fibers exist. However, as the blue fiber has a branching ratio, the input tree of the yellow fiber also branches out. The corresponding complete building block of structure **G** is also shown to illustrate how such a complex and big structure could be reduced to a much simpler and more understandable structure at its base.

All Fibonacci structures such as simple Fibonaccis, Composite FeedForward and Composite Feedback Fibonaccis are characterized by a branching input tree. By only specifying the branching ratio of a building block, we do not determine the type of class of the building block. In fact, two building blocks can have the same branching ratio but can belong to different classes, while a simpler class can have a higher branching ratio than a more complex class. For example, in Fig. 4, building block E corresponds to a simple Fibonacci building block, yet features a higher branching ratio (at r=3.79) than blocks B and C, even though both are examples of Composite Fibonacci building blocks.

#### Composite Feedforward Fibonacci building blocks

A Composite Feedforward Fibonacci is a multilayer building block with a branching input tree, the key feature of Fibonacci fibers. Such a structure occurs when a fiber in a Fibonacci structure regulates (an)other fiber(s) downstream in a feedforward manner. The Fibonacci fiber-regulator as part of a Fibonacci structure has a branching input tree, that in turn gets “passed along” to the nodes it regulates. In other words, the Fibonacci fiber “inherits” and passes its input tree to the regulated fiber, causing the regulated fiber’s input tree to branch out.

As a complex structure in Fig. 3C, yellow nodes *mmuM* and *metE* only receive input from both *metC* and *malY*. Focusing on *metC* and *malY* we observe that both nodes are embedded in a Fibonacci structure through their regulator *tnaa*. As for the yellow fiber *mmuM* and *metE* we find that the input tree of the blue fiber is “inherited,” thus prompting its own input tree to branch out as well.

#### Composite feedback fibonacci building blocks

A Composite Feedback Fibonacci occurs when the feedback loop (or loops) of a Fibonacci structure crosses two or more fibers, as shown in Fig. 3D. When we construct the input tree for the blue fiber *gsk-nepI*, we observe that we need to include the nodes in the yellow fiber *xapA-ppnP-deoD* as well, since they act as regulators of the blue fiber. In turn, the nodes of the blue fiber regulate the yellow fiber, thus creating a loop between them. As a consequence of the loops between these fibers, the blue fiber also acts as a regulator to the yellow fiber. This loop causes the input tree to branch out as $a_{i}=1,5,11,26,137,314,\ldots$, pointing to a branching ratio of r=2.3.

As a consequence, one fiber cannot be defined without the other(s), as each fiber acts as a regulator for the other fiber(s), suggesting that the separation of a regulator from the other fiber is impossible. As these structures can connect multiple fibers, multiple fibers in the simplest possible case are linked in a ring-like cycle where each fiber is only connected to two others (i.e. one by an input and one by an output). In turn, all the fibers can directly be linked to each other, resembling an almost fully connected graph. Distinguishing these two structures, only one feedback loop (i.e. one single cycle of length *n*) encompasses all fibers in the first case while a multitude of different feedback loops exist in the other case. In more detail, in a base that connects *n* fibers in such a fully connected scheme the total number of cycles corresponds to $\left(\begin{array}{c} n\\ 2 \end{array}\right)$ number of cycles of length 2 plus $\left(\begin{array}{c} n\\ 3 \end{array}\right)$ cycles of length 3 and so on up to one cycle of length *n* (without accounting for external regulators), pointing to a higher branching ratio.

As a consequence of the dense connectivity of these networks, the observed *Composite Feedback Fibonacci* structures are closer to the latter than the former case. We find such a structure in the oxidative enzyme network at the bottom of Fig. 4, where the base of the structure shown at the bottom right corner is almost a fully connected network encompassing 5 different fibers and 4 regulators that participate in the feedback loops (plus another regulator not included in any loop). As a result we find a complex structure, pointing to an ‘entanglement’ of numerous cycles and connecting several fibers with a high branching ratio. All enzyme networks exhibit this type of arrangement at the center of the network, as shown in Fig. 4 in the oxidative network. Such structures present the highest branching ratios, with the amino-acid network indicating one such structure with r=12.39 and another with r=25.97 while the carbon network provides one with r=10.81, the glycolysis network with a r=13.65, and the oxidative network with r=10.37.

As a corollary, these two composite Fibonacci structures can be combined, when a fiber in a *Composite Feedback loop* further regulates—albeit in a feed-forward manner—other fiber(s) as shown with the fibers regulated outwardly from the central *Composite Feedback* structure in the oxidative network at the bottom of Fig. 4.

Lastly, a consequence of considering only *shortest* cycles to construct building blocks lies in a fiber that might be a part of more than one building block. Such a situation occurs when longer cycles that cross (an)other fiber(s) are not included in a fiber’s building block since they are longer than the shortest cycle between fibers and regulators. For example, the addition of a second fiber to a simple Fibonacci building block (r=1.618) regulates the first fiber and the only external regulator. This addition creates a new cycle of length 3 that includes both fibers. When the building block of the original first fiber is constructed, this new cycle is ignored since it is longer than the shortest cycle between the original fiber and its external regulator. However, when constructing the building block of the second fiber, the original fiber needs to be included since it regulates this newly added fiber. As a result, this second building block does include the longer cycle and both fibers, resulting in a Composite Feedback Fibonacci building block.

In such cases, we consider the smaller building block the “*defining*” one for the fibers included in both, since the shortest cycles have a dominant effect on the dynamics of this fiber or fibers. The bigger building blocks are naturally the “*defining*” ones for the fiber(s) only included in it, while it can be considered a form of “higher-order” correction on the dynamics of the fiber(s) present in both building blocks.

### Complexity Ladder of branching ratio *r*: Effect of cycles on Fibonacci fibers

The wide range of branching ratios found in metabolic networks ranging from r=1.618 to r=25.97 prompts us to understand the ways in which addition or changes of cycles influence the complexity of the circuits. Figure 4A–G presents different examples of observed single and composite Fibonacci building blocks and their place in such a complexity landscape. The input tree is characterized by the sequence $a_{i}$ of enzymes in the *i*th generation. As for Fibonacci fibers this sequence can often be described by a close form recurrence relation, such as $a_{i}=a_{i-1}+a_{i-2}$ as observed with most basic Fibonacci fibers. In particular, the first term ($a_{i-1}$) represents a self-loop, a cycle of length of 1, while the second term ($a_{i-2}$) represents a cycle of length 2 between the fiber and the regulator. Generally, a cycle of length *d* contributes a term $a_{i-d}$ to the recurrence relation.

Specifically, the increase of the number of cycles with the same length changes the multiplicity of their terms in the recurrent relation of $a_{i}$, thus increasing the branching ratio *r*. In particular, multiple *n* cycles of the same length *d* contribute the term $na_{i-d}$ to the series $a_{i}$. The addition of nodes and edges that contribute more cycles, as shown in Fig. 3D, increases the number of nodes in further generations of the $a_{i}$ sequence, prompting an increase of the branching ratio *r*. The value of their contributions varies depending on the length of the additional cycles or the change in multiplicity to the edges in an existing cycle.

Figure 3B shows the ways a branching ratio of r=2.41 can be obtained from the simplest Fibonacci structure by adding cycles. In particular, the simplest Fibonacci structure consists of a regulator and the fiber itself, together with a feedback loop that connects the two nodes as well as an autoregulation loop on one of the nodes. The addition of more regulators does not change the branching ratio unless they contribute more cycles. In turn, the addition of edges that create more cycles between regulators and the fiber increases the branching ratio.

A cycle of length 3 that connects the fiber and the two regulators, changes the sequence to $a_{i}=a_{i-1}+a_{i-2}+a_{i-3}$, pointing to a branching ratio increase from r=1.62 to r=1.84. Adding another cycle of length 1, which can be done two different ways, gives the resulting r=2. By combining the two separate cycles with the regulators and adding another edge connecting the regulators—resulting in the same length cycle of length 3 as before—we obtain $a_{i}=a_{i-1}+2a_{i-2}+a_{i-3}$ with r=2.15. Lastly, including all possible cycles in this 3-node structure results in $a_{i}=2a_{i-1}+a_{i-2}$.

To increase the branching ratio of a building block, the number of cycles needs to be increased which can be achieved by changing the multiplicity of the edges in the structure. For example, the block in Fig. 4G essentially has the same basic structure as the simplest Fibonacci structure in Fig. 4A. However, the multiplicity of the edges is higher, indicated by 4 directed edges that connect the yellow fiber to the blue fiber. In turn, 6 directed edges link the blue with the yellow fiber while the self-loop also has a multiplicity of 6, resulting in a structure with a highly elevated branching ratio of r=8.75. Alternatively, one can add more nodes and edges in such a way that the number of cycles is increased, as shown in Fig. 3B. In the case in which the added nodes form a part of a fiber, the added cycles would in fact create a Composite Feedback Fibonacci, since they connect fibers through a feedback loop.

When studying the structure of these networks (Table 1), we find that a lot of the nodes in fibers belong to SCCs, a natural consequence of having both a high coverage of nodes that belong to fibers, as well as a large percentage of nodes in the network belonging to SCCs. These two facts make the perfect scenario for the existence of feedback loops connecting multiple fibers, which point to very complex Composite Feedback Fibonaccis with several fibers. We observe this case in the building block at the center of the oxidative enzyme network (depicted at the bottom of Fig. 4), where we found 5 fibers and 4 regulators entangled in a myriad of cycles between them. Such structures actually present the biggest branching ratios through an elevated number of different cycles to connect multiple fibers together with a set of regulators. For example, the building block at the center of the oxidative enzyme network (Fig. 4) exhibits a branching ratio of r=10.37.

### Characterization of fibers

**Fig. 5.**
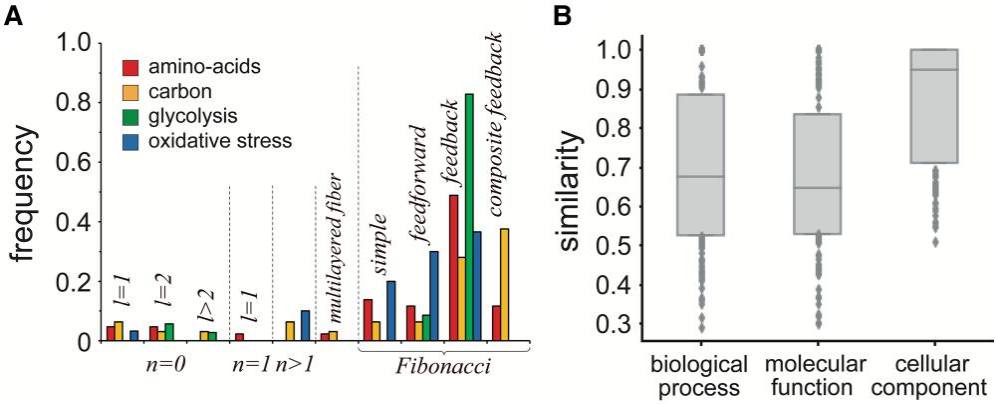
Biological significance of fibers. A) As for the presence of different types of fibers in the amino-acid synthesis, carbon metabolism, glycolysis and oxidative stress networks, Fibonacci fibers are most abundant in all networks. B) Fibers show high average functional similarity in the underlying four enzyme networks.

The landscape of fibration building blocks in the studied metabolic networks is dominated by highly complex Fibonacci structures (Fig. 5A). This observation becomes apparent when we compare our four enzyme networks with the TRN of *E. coli* (14, 21), where we only observed simple Fibonaccis. In principle, the whole network structure of the four enzyme networks is drastically different compared to the TRN that is composed of six strongly connected components with roughly only 2% of all nodes belonging to any of the SCCs (21). In contrast, in the metabolic network, the number of nodes that belong to SCCs ranges from around 47%, in the oxidative network, to close to 62% in the carbon network (Table 1). The typical structure of enzyme networks exhibits a notable pattern in that they tend to have one significantly large SCC, covering a substantial portion of the entire network, alongside a few smaller SCCs.

Furthermore, the enzyme networks are also more densely interconnected. For instance, the average degree ranges from 5.79 in the oxidative network to 11.72 in the amino-acid network, whereas the TRN has an average degree of 1.94. As a consequence of higher connectivity a substantially higher number of cycles in these networks exist, providing necessary cycles to obtain highly complex Fibonacci structures.

One of the most apparent differences between the simple Fibonaccis found in the TRN and the complex Fibonaccis in the enzyme networks is the range of their branching ratios. In the TRN, we observe only three Fibonaccis with a branching ratio ranging between 1.38 and 1.61, while in the enzyme networks we observe values almost as high as 26. Such an observation is mainly a consequence of the increased number of cycles in these networks, ensuring multiple different possible ways to establish cycles between the fibers drastically increasing their complexity.

### Biological significance of fibers

Characterizing the topological features of the fibration building blocks, we have shown that increasingly larger cycle arrangements provide high complexity to their structures, yet, in a well structured way leading to a systematic classification of their structures (Fig. 5A). To test the significance of these complex building blocks for the functionality of the cell, we consider the functional similarity of enzymes in fibers, hypothesizing that the participation in synchronized groups of enzymes also translates into functional similarity. In particular, we determine the mean similarity of gene ontology (GO) terms using GoSemSim (26) over all pairs of enzymes in a fiber (Fig. 4B). We observe that enzymes in fibers are highly similar in their functions when we consider the molecular function, biological processes and cellular components GO categories (Fig. 5B). Confirming such patterns, we find similar functional similarity of enzymes in fibers in each enzyme network, separately (Fig. S2).

**Fig. 6.**
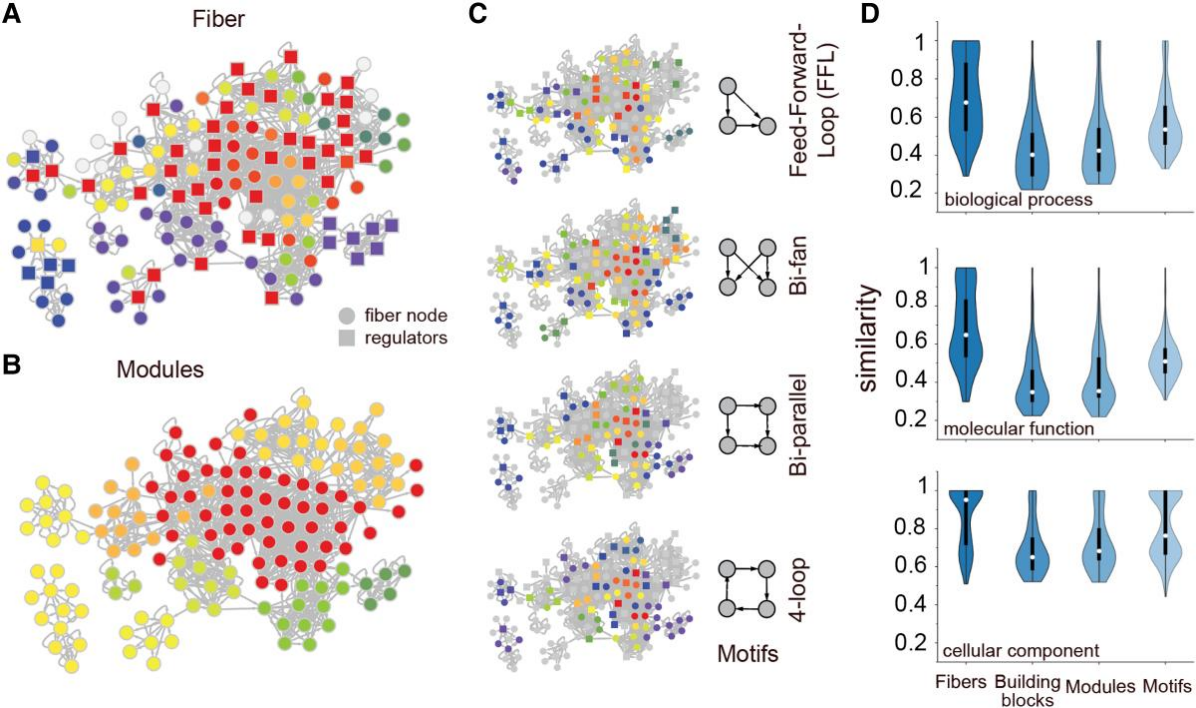
Fibers, modules, and motifs I. In A), we determine fibers in the carbon enzyme network, where colors refer to proteins in the same fiber. In B), we determine topological clusters using the Louvain algorithm, while C) points to the presence of significant motifs of different types. D) Violin plots of the average functional similarity of enzyme pairs in fibers, clusters, motifs and fibration building blocks using GO terms indicate that enzymes in fibers significantly show higher similarities compared to enzymes in clusters, motifs and building blocks ($P<10^{-3}$, Mann–Whitney *U* test).

The overwhelming presence of fibration building blocks in the enzyme networks prompts us to compare their characteristics and biological significance to the standard way to calculate building blocks using network motifs and modules (or community structure) (Fig. 6A–C). Focusing on the carbon metabolism subnetwork of enzymes, we determine modules using the Louvain algorithm (18), and search for the most popular network motifs, such as feed-forward loops, Bi-Fan, Bi-parallel and 4-cycle motifs (3, 4).

A visual inspection of the coverage of fibers, modules, and motifs in Fig. 6A–C indicates that modules partition nodes in the underlying networks, while fibers and motifs expectedly cover the underlying network partially. In particular, the determination of modules and motifs is based on connectivity in the underlying network while fibers account for the similarities of input trees. As a consequence, motifs and modules merely reflect local connectivity. In contrast, fibers bundle genes that share the same information flow, suggesting that synchronicity transcends simple connectivity. In other words, nodes can be synchronized (i.e. share the same input tree) without being necessarily connected in contrast to motifs and modules.

Given that fibers bundle information flow, we hypothesize that functional roles of enzymes in the same fiber show a higher degree of functional similarity than motifs and modules that are based on statistical significance and simple connectivity. As a benchmark, we compare functional similarity of fibers with motifs and modules, given that strongly interacting proteins have a heightened propensity to be involved in similar biological functions. In Fig. 6D, we observe that the average functional similarity of enzyme pairs in fibers is higher than their module and motif counterparts in all enzyme networks using GO terms from all three ontologies. We find similar results when we consider fibers, modules and motifs in all metabolic networks separately (Fig. S3). We further considered the functional similarity of building blocks that in the most complex way are composed of many fibers, such as composite feed-forward and feedback Fibonaccis. As for their functional similarity, we still find that building blocks have similarities that compare to modules (Fig. 6D).

**Fig. 7.**
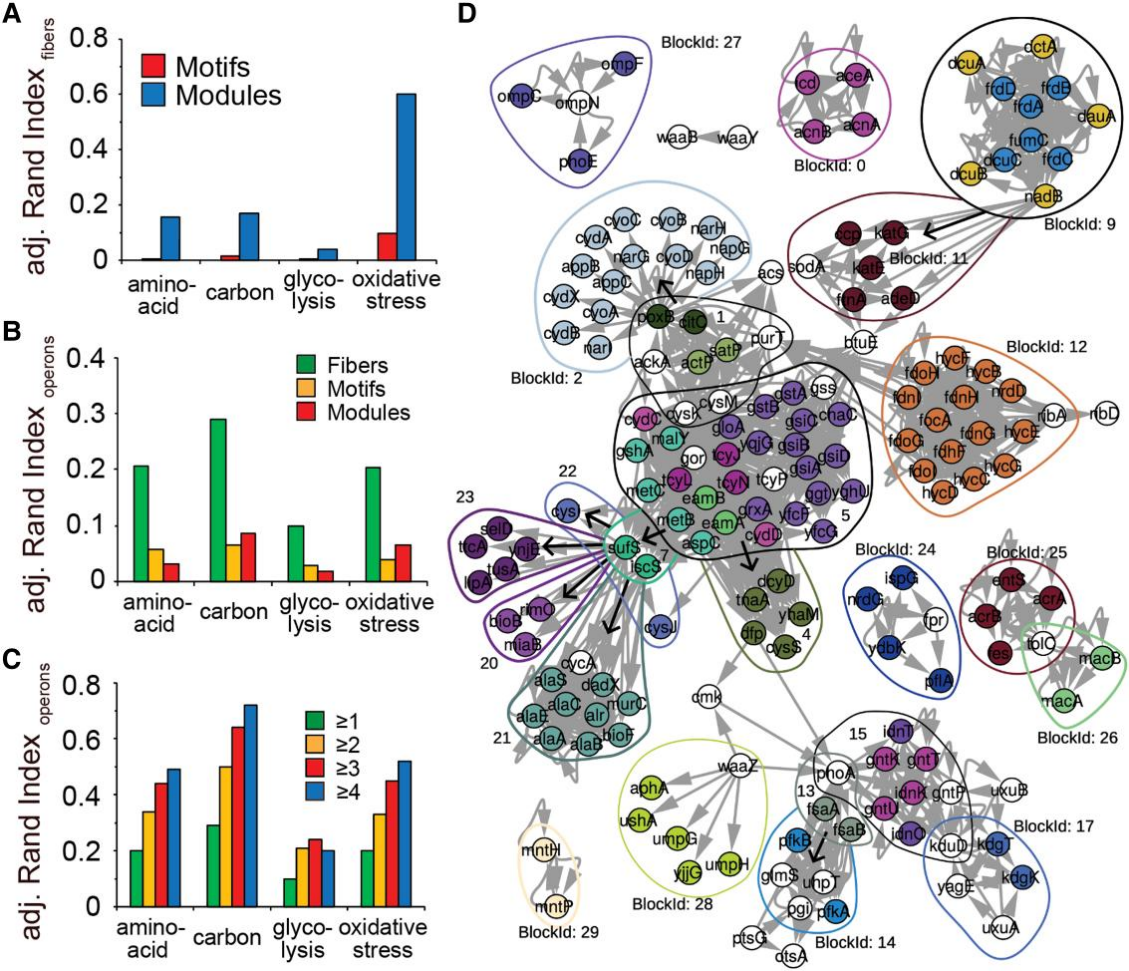
Fibers, modules, and motifs II. A) Determining an adjusted Ward index as a measure of the similarity of two partitions, we found that fibers are better overlapping with modules than motifs in all enzyme networks. In B), we determine the similarity between genes in operons and fibers, modules and motifs, indicating that fiber gene sets show the greatest overlap with operon gene sets. In C), we observe that the propensity of fibers in the underlying metabolic networks to overlap with genes in operons increases with elevated operon size. In D), we show a map of building blocks (enclosed by closed lines) and their constitutive fibers (nodes in color) in the oxidative stress network. Note, that some regulators may belong to more than one building block, while the fibers are disjoint.

Furthermore, we wonder to what extent fibers, modules and motifs complement each other. Determining an adjusted Rand Index, we find that modules overlap significantly better with fibers than motifs in all four metabolic networks (Fig. 7A). Such an observation is rooted in the fact that modules arise from partitioning all nodes in an underlying network, therefore increasing the chances that large modules harbor comparatively small fibers. In turn, hardly all nodes participate in at least one motif or fiber, diminishing the chances that such structures substantially overlap.

As another indication of the biological relevance of genes that are organized in fibers, we determine the overlap with genes that appear in operons in *E. coli*, assuming that symmetries that dominate the network topology are reflected in the regulatory organization of gene sets. Utilizing 2,579 operon gene sets from the RegulonDB databases (20), we determine overlaps with an adjusted Rand Index and find that fibers overlap significantly better with operons than motifs and modules in all four different metabolic networks (Fig. 7B). Furthermore, we refine our analysis by considering operons that consist of at least a certain number of genes, indicating that fibers increasingly overlap with operons that harbor more genes (Fig. 7C).

In Fig. 7D, we mapped fibers and building blocks in the oxidative stress network. As an example, we observe a fiber that is composed of the *fduABCD* operon of genes building the fumarate reductase enzyme complex (BlockId 9, Fig. 7D). Such a fiber is entangled with another fiber in a complex feedback loop that is composed of subunits of the anaerobic C-4-dicarboxylate transporter *d*cuABC and *d*ctA, that is responsible for the uptake of fumarate, succinate, L-aspartate and L- and D-malate under aerobic conditions. While the underlying connections between the single enzymes that are involved in the transport of metabolites and corresponding reductase are highly complex, we break the underlying complexity down to two fibers that not only bundle the underlying functions effectively but also indicate which functions are connected to each other on a dynamic basis. In other words, the complex multiplicity links between enzymes are mapped to relatively simple building blocks of enzymes that are dependent on each other, pointing to the relevance of fibers as representatives of potential pathways.

## Discussion

Focusing on metabolic networks, we identify symmetries between enzymes that resulted in fibration building blocks. Branching ratios of the fibers are overwhelmingly noninteger numbers, pointing to the presence of complex Fibonacci fibers. Such structures feature cycles between the fiber and at least one of the regulators, ensuring that information is sent back from the fiber to the regulator and back to the fiber. Although some of the simplest forms of these Fibonacci fibers have already appeared in the *E. coli* TRN (14, 17), we find that the metabolic network is rich in novel forms of more complex structures. In particular, the addition of edges that lead to the emergence of cycles increases the branching ratio substantially. Furthermore, we detect complex Fibonaccis, where one fiber cannot be defined without the other(s), suggesting that each fiber acts as a regulator for the other fiber(s). We find that the highest level of complex fractal input trees are structures that occur when the regulator of a fiber in a Multi-Layer structure itself is a Fibonacci structure. Such observations indicate that regulators are not limited to single nodes (as observed in the TRN) but also can be larger, complex fiber structures.

Links between enzymes in fibers are metabolic passage ways, pointing to a flow of information that is exchanged through metabolites that enzymes work on in concerted reactions. The ensuing network of enzymes in consecutive reactions features a highly complex topology. Our fibration analysis allows us to bundle enzymes with the same input trees and impose a hierarchical order of complexity. As a consequence of bundling enzymes in complex fibers, such interplay between fibers provides a glimpse into their stability and robustness.

In our example in Fig. 7D, we observe that a complicated web of interactions between components of the *fduABCD* operon and subunits of the anaerobic C-4-dicarboxylate transporter *d*cuABC and *d*ctA, was reduced to a relatively simple feedback Fibonacci fiber that featured two fibers, referring to the fumarate reductase enzyme complex and the transporter. Moreover, such a Fibonacci fiber featured numerous feedback links between the two sub-fibers suggesting that the structure still prevails if a connection vanishes. In other words, while the multiplicity of links may change as the consequence of e.g. a mutation in an underlying enzyme the fiber structure is still kept, suggesting the robustness of the building blocks.

As a corollary, such fibers bundle metabolic information as elementary biologically relevant building blocks. To lend biological credence to fibers, we show that enzymes in fibers share similar functions as the concept of a topological fiber points to groups of nodes that work in a concerted way. Indeed, we observe that enzymes that are organized in fibers show a high level of functional similarity.

To compare to other elementary building blocks of biological networks, we consider network motifs and modules. As a consequence of the fundamentally different ways of their detection, we observe that motifs and modules are significantly less functionally similar than fibers, suggesting that coherence between genes is a better indicator of biological significance than the over-representation of topological patterns. Furthermore, motifs fail to complement fibers, while network modules show the opposite. Such a finding is probably rooted in the observation that modules partition nodes in the network, increasing the chances that such groups of nodes harbor fibers as well. Furthermore, the underlying fibers are bundles of enzymes where metabolic information flows in a coherent way, suggesting that such patterns may also be reflected in the genomic arrangements of genes in operons and fibers in the transcriptional regulatory network. Indeed, we find that the overlap between fibers is better established than motifs or modules, suggesting that fibers point to biologically relevant, elementary building blocks.

Such fibers that potentially act as elementary building blocks of metabolic networks may also correspond to extreme pathways (27) and elementary flux modes (28). Extreme pathways are a unique set of systemically independent biochemical pathways that are based on system stoichiometry. Such pathways represent the edges of the steady-state flux cone derived from convex analysis that can be used to represent any flux distribution in the underlying metabolic network. Furthermore, elementary flux modes are a mathematical tool to define and comprehensively describe all metabolic routes that are both stoichiometrically and thermodynamically feasible for a group of enzymes. As a consequence, such methods allow us to find paths in the network, depending on the underlying metabolic objective. Although our analysis of the underlying metabolic network of *E. coli* did not account for any stoichiometric characteristics and considers the underlying metabolic network as a whole it is conceivable that fibers may be building blocks of such extreme pathways and elementary flux modes as well. In particular, we surmise that groups of fibers could be modules, allowing an explanation of the regulation of the corresponding extreme pathways and flux modes.

Although our underlying metabolic network was compiled from the latest updated sources and portrays the most complete set of metabolic reactions, we surmise that such information still suffers from a degree of incompleteness. While we cannot rule out that the absence and/or presence of spurious links may impact our results, we observed that more complex fibers show an inherent degree of stability. For example, we found that more complex Fibonacci fibers are entangled in a web of multiple feedback loops between the same bases (Fig. 4G). As a consequence, the presence of spurious links in the underlying network would not impair the underlying structure of the fiber, pointing to a level of stability toward missing links that is fed by the structure of the underlying metabolic network. As a consequence, we surmise that the experimental knock-out of a corresponding enzyme would not destroy the underlying fiber. Instead, the resilience of the fiber may translate into the robustness of the underlying metabolic activity that could be experimentally tested.

As another aspect of the incompleteness of the underlying metabolic network we stress that our analysis of the symmetric properties is based on a skeleton of metabolic reactions where we dis-account for reactions that change their directionality dynamically as a result of concentration changes. We surmise that such changes in the underlying topology could have an impact on symmetric features. In particular, we expect that such characteristics may change the structure of fibers as dynamic building blocks that reflect metabolic characteristics of such reactions.

In our approach, we assume synchronous dynamics and the same timescale for all dynamics on the network. However, in protein–protein interactions, some proteins interact synchronously with others, while some appear to act asynchronously (29), suggesting that also unsymmetric relationships may exist. However, we find that the majority of nodes in the underlying network are indeed organized in fibers, suggesting that the underlying symmetry assumption is indeed dominating the network.

We conclude that fibers are plausible elementary building blocks that bundle metabolic information in metabolic pathways, potentially pointing to a novel way of considering metabolic dynamics on a topological level. While fibers and modules are simply considering the local connectivity of their underlying nodes, fibers capture the “history” of metabolic information flowing into them. In other words, the underlying, complex dynamics of the network is better captured through input trees than simply evaluating the over-representation of motifs or partitioning of nodes in the complete network. This opens the doors to a systematic characterization of biological pathways with significance to find novel drug targets as points of therapeutic intervention.

## Materials and methods

### Metabolic networks

We constructed metabolic networks by utilizing enzyme information from the “All Enzymes of E. coli K-12 Substrate MG1655” data set as of the EcoCyc database (19). Utilizing information of metabolic reaction that are catalyzed by such enzymes from RegulonDB (30), we connected genes, when the product of one reaction that was catalyzed by an enzyme is the substrate for a subsequent reaction that is catalyzed by a different enzyme.

To construct four function-specific metabolic networks, we establish relevance of metabolic reactions by the presence of transcription factors that controlled the expression of corresponding enzymes as of RegulonDB (30). In particular, we filter metabolic reactions through enzymes that were regulated by transcription factors expressed during oxidative stress from (31). In the same way, we extract transcription factors that are associated with carbon sources from the RegulonDB Sensor Units Datasets (30). In turn, we derived metabolic reactions that pertain to aminoacid metabolism from genes related to a specific growth condition obtained from (32). As for the glycolysis network, we filtered all metabolic reactions if they were catalyzed by enzymes that appeared in the corresponding KEGG pathways (33).

Moreover, we stress that various ubiquitous metabolites such as $H_{2}O$, ATP, etc. exist. To curb their influence, we sorted all metabolites in each subnetwork according to their occurrence in the underlying metabolic reactions and manually discarded most occurring metabolites.

### Minimal balanced coloring algorithm

While several algorithms are used to find fibers in networks (22, 23), all available algorithms are based on finding “balanced equivalence” relations in the network (see (15) for details). Current algorithms are based on the algorithm introduced by Cardon and Crochemore (23). Here, we used the version developed by Kamei and Cock (24). A detailed explanation of this algorithm is given in Ref. (14). In a recent review article, we further discuss a fast algorithm that is scalable to large system sizes in Ref. (34). The code of the algorithm in R can be accessed at https://github.com/ianleifer/fibrationSymmetries.

### Algorithm for determining branching ratio

To determine the branching ratio of any input tree *r*, we need to determine the sequence $a_{i}$ first that is the number of nodes present in the *i*th layer of the input tree. Specifically, in our approach we consider the adjacency matrix of the structure in question (**adj**) as well as a specified node (**j**). We determine the sequence $a_{i}$ and the branching ratio *r* of the specified node by iteratively calculating the number of times each node is present in the *i*th generation of the input tree, based on the input relations from the adjacency matrix **adj** and the number of times each node appears in the previous generation i−1. The algorithm then calculates $a_{i}$ by summing the number of nodes present in that generation. After this step, the algorithm computes *r* by calculating $\frac{a_{i}+1}{a_{i}}$. This process is iteratively repeated until the value *r* does not change compared to the previous value.

The algorithm begins by initializing two vectors: the sequence vector **seq** (which stands for the sequence series $a_{i}$) and a vector of vectors called **inp tree**. Each entry in **inp tree** is a vector, the size of which is equal to the number of nodes in the network (taken from the dimensions of the given adjacency matrix) while each entry in this vector represents one of the nodes of the network. The vector **inp tree** stores the number of times each node appears in the *i*th generation of the input tree, represented by the *i*th entry in **inp tree**. The algorithm starts with just one entry in **inp tree**, the first generation, and begins by making the entry corresponding to node **j** equal to 1 (this is the “root” of the input tree) and the entry for all other nodes 0. Then, based on the input relations from **adj**, determines how many times each node sends an edge to **j** and updates the values of the i+1 entry of **inp tree** accordingly. The sum of the values in the *i* entry of **inp tree** is used to determine the *i*th entry of the sequence vector **seq**. Then the value of **r** is calculated by dividing the last entry in **seq** with the second-to-last entry. This process is then repeated with the last entry of **inp tree** being the starting point to trace back the inputs and then obtain the new entry in both **inp tree** and **seq**. This is repeated until the value of **r** is the same compared to the previous iteration.

### Adjusted Rand index

The Rand index allows the assessment of the similarity between two clusterings of same data points (35). In particular, the adjusted Rand index is the corrected-for-chance version of the Rand index, establishing a baseline by using the expected similarity of all pairwise comparisons between clusterings specified by a random model. Given a set *S* of *n* elements, and two groupings of these elements $X=\{X_{1},X_{2},\ldots ,X_{r}\}$ and $Y=\{Y_{1},Y_{2},\ldots ,Y_{s}\}$, the overlap between *X* and *Y* can be summarized in a contingency table $\left[n_{ij}\right]$ where each entry $n_{ij}$ is the number of common elements in groups $X_{i}$ and $Y_{j}$, $n_{ij}=|X_{i}\cap Y_{j}|$. The original Adjusted Rand Index using the Permutation Model is defined as


(4) $$\mathrm{ARI}=\frac{\sum_{ij}\left(\begin{array}{c} n_{ij}\\ 2 \end{array}\right)-\left[\sum_{i}\left(\begin{array}{c} a_{i}\\ 2 \end{array}\right)\sum_{j}\left(\begin{array}{c} b_{j}\\ 2 \end{array}\right)\right]/\left(\begin{array}{c} n\\ 2 \end{array}\right)}{\frac{1}{2}\left[\sum_{i}\left(\begin{array}{c} a_{i}\\ 2 \end{array}\right)+\sum_{j}\left(\begin{array}{c} b_{j}\\ 2 \end{array}\right)\right]-\left[\sum_{i}\left(\begin{array}{c} a_{i}\\ 2 \end{array}\right)\sum_{j}\left(\begin{array}{c} b_{j}\\ 2 \end{array}\right)\right]/\left(\begin{array}{c} n\\ 2 \end{array}\right)},$$

where $a_{i}=\sum_{j}n_{ij}$, $b_{j}=\sum_{i}n_{ij}$ and *n* is the number of all data points.

### Louvain clustering

Using a greedy agglomerative procedure, the Louvain clustering algorithm maximizes the modularity *Q* of a partition $C=C_{1},\ldots ,C_{p}$ of a directed graph *G* defined as


(5) $$Q=\frac{1}{m}\sum_{u,v}[E_{uv}-\frac{d_{u}^{in}d_{v}^{out}}{m}]\delta (C_{u},C_{v}),$$

where m=|E| is the number of edges of G, $E_{uv}$ represents the existence(0 or 1) of an edge from community *u* to community *v*, $d_{u}^{\mathrm{in}/\mathrm{out}}$ represents in/out degrees of community *u* (18).

### Motif detection

To establish the statistical significance of the raw number of subnetworks of size $N_{i}$ of a given node *i*, the underlying network topology is scrambled by randomly distributing the same number of links keeping the degree of nodes unchanged. To assess the significant presence of a given motif in the scrambled networks, we determine the mean number $\left\langle N_{i}\right\rangle$ and SD $\sigma_{i}$ to calculate a Z-score $Z_{i}=\frac{N_{i}-\langle N_{i}\rangle }{\sigma_{i}}$ for each motif. In particular, we consider motifs *i* as significantly appearing if $Z_{i}>2$ (3, 4). Furthermore, we account for motifs of length 3 and 4, as such small motifs are most comparable to the size of fibers.

## Supplementary Material

pgaf080_Supplementary_Data

## Data Availability

The data and methods underlying this article are available in https://github.com/makselab/Symmetries-in-Metabolic-Networks-of-E.-coli and https://osf.io/2ntr3/.
