# Supplementary material for: Symmetries in metabolic networks of Escherichia coli
Source: PNAS Nexus. 2025 Mar 17;4(3):pgaf080. doi: 10.1093/pnasnexus/pgaf080 (PMC11937945; doi:10.1093/pnasnexus/pgaf080)
Supplement: pgaf080_Supplementary_Data [file pgaf080_supplementary_data.pdf]

Symmetries in metabolic networks of *E. coli* - Supplementary Material

Luis Alvarez[1†], Kuang Huang[1†], Cecilia Ishida[2], Mishael Sánchez-Pérez[3], Stefan Wuchty[4,5,6,7], Hernán A. Makse[1*]

[1]Levich Institute and Dept. of Physics, City College of New York, New York, 10031, NY, USA.
[2]Faculty of Medicine and Biomedical Sciences, Autonomous University of Chihuahua, Chihuahua, 31125, Mexico.
[3]Centro de Ciencias Genómicas, Universidad Nacional Autónoma de México, Cuernavaca, 62210, Mexico.
[4]Dept. of Computer Science, Univ. of Miami, Coral Gables, 33146, FL, USA.
[5]Dept. of Biology, Univ. of Miami, Coral Gables, 33146, FL, USA.
[6]Institute of Data Science and Computing, Univ. of Miami, Coral Gables, 33146, FL, USA.
[7]Sylvester Comprehensive Cancer Center, Univ. of Miami, Miami, 33136, FL, USA.

[†]These authors contributed equally to this work.

Supplementary Figures and Tables

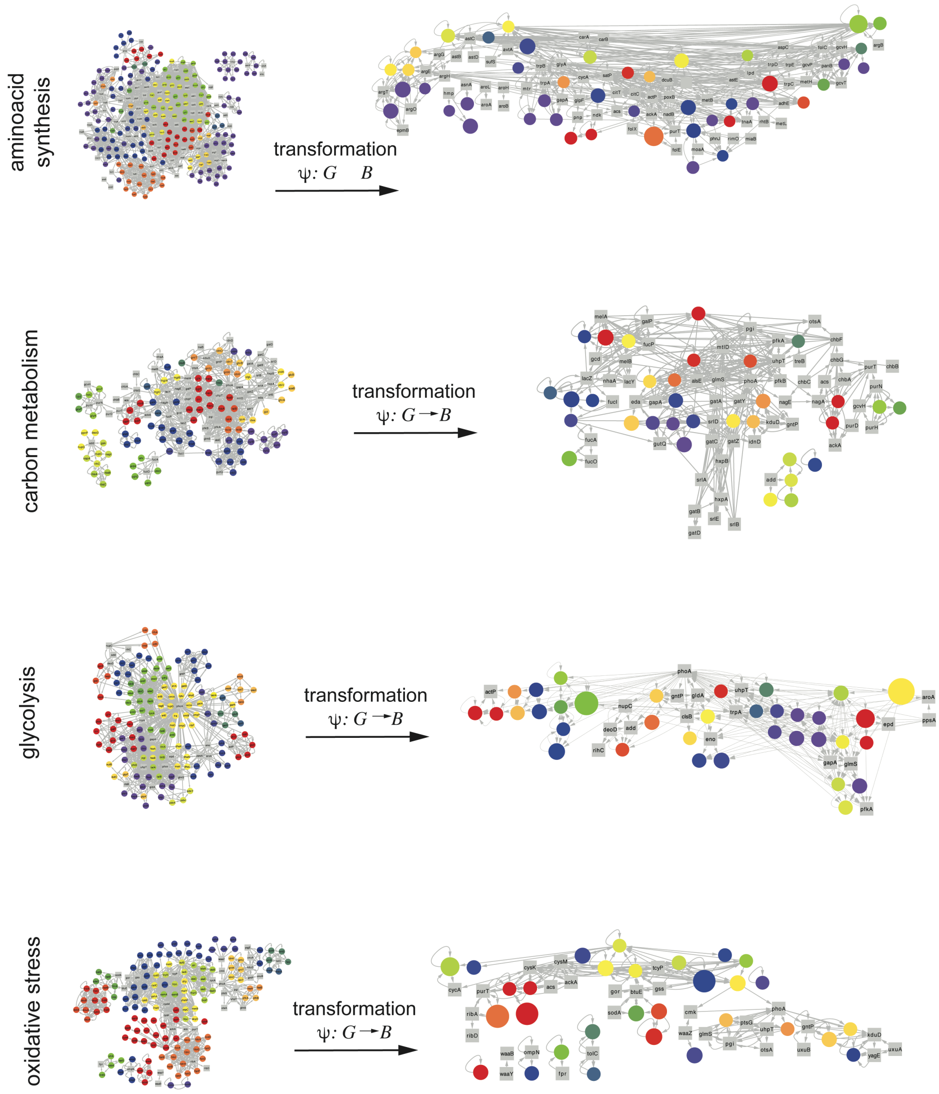

Fig. 1 Fibers in the aminoacid synthesis, carbon metabolism, glycolysis and oxidative stress enzyme network. We determined fibers in these enzyme networks in *E. coli*, where colors refer to synchronized enzymes. Furthermore, we collapsed the nodes in a fiber into one large node, transformed the whole networks G into its bases B.

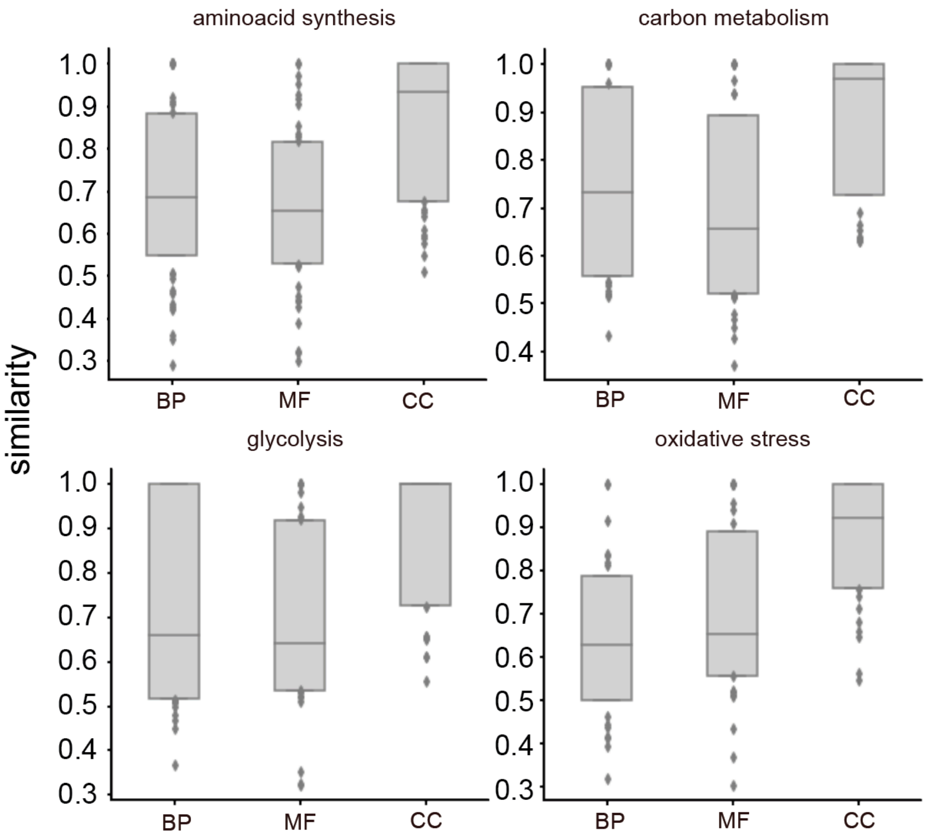

Fig. 2 Functional similarity of fibers in the aminoacid synthesis, carbon metabolism, glycolysis and oxidative stress enzyme network. In each subnetwork, we calculated the mean similarities of all enzyme pairs in the fibers and their regulators. In all GO ontologies including the molecular function (MF), biological processes (BP) and cellular components (CC) ontologies and subnetworks, we clearly observe that enzymes in fibers are functionally highly similar. As expected, when we include the regulators of the fibers in the analysis, we find that they are not as functionally homogeneous as the enzymes within fibers. This is because the regulators most of the time are master regulators participating in different fibers and therefore in different functions.

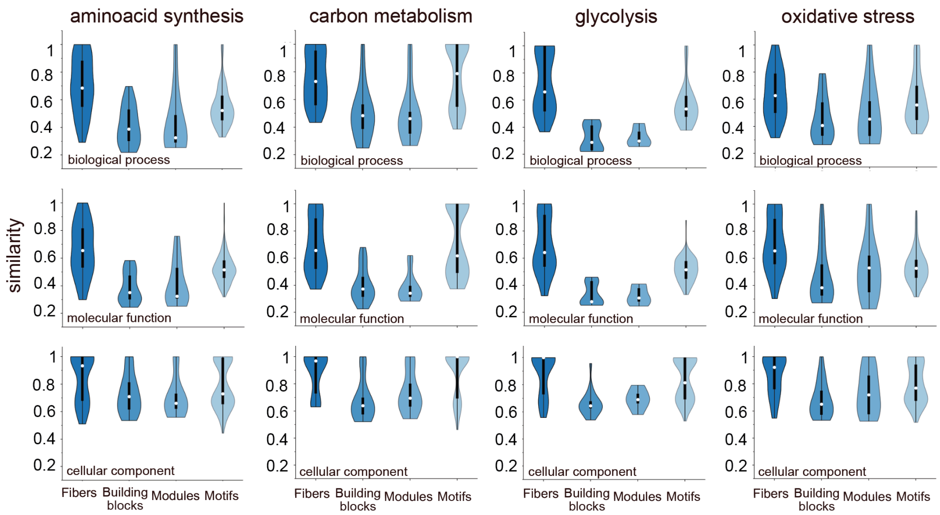

Fig. 3 Functional similarities of fibers, clusters and motifs in the aminoacid synthesis, carbon metabolism, glycolysis and oxidative stress enzyme networks. Violin plots refer to the average functional similarity of fibers, clusters, motifs and building blocksusing GO terms from the molecular function (MF), biological processes (BP) and cellular components (CC) ontologies. Largely, we observe that fibers significantly show higher similarities compared to clusters, motifs and building blocks ($P < 10^{-3}$, Mann-Whitney U-tests).

Table 1 Topological characteristics of the aminoacid synthesis, carbon metabolism, glycolysis and oxidative stress enzyme networks

network	N_{nodes}	N_{edges}	N_{fibers}
aminoacid synthesis	261	3,059	44
carbon metabolism	149	933	32
glycolysis	153	973	35
oxidative stress	168	972	30

Table 2 Topological characteristics of the base representation of aminoacid synthesis, carbon metabolism, glycolysis and oxidative stress enzyme networks

network	N_{nodes}	N_{edges}
aminoacid synthesis	107	603
carbon metabolism	86	395
glycolysis	53	260
oxidative stress	61	205

Table 3: List of fibers in amino acid network

Fiber Id	Nodes in Fiber	Class
14	spoT, nudJ	FF Fibonacci
16	nrdB, nrdA, nrdF, nrdE	FF Fibonacci
23	cysK, cysM	FB Fibonacci
26	dauA, dctA, dcuA	FB Fibonacci
28	dcuC, frdD, frdC, frdB, frdA, sdhA, sdhB, sdhC, sdhD	FB Fibonacci
30	scpC, sucC, sucD	FB Fibonacci
35	ilvH, ilvI, ilvN, ilvB, ilvM, ilvG, pflB, tdcE	Fibonacci
36	metC, malY	FB Fibonacci
37	adiC, argS, artP, artM, artQ, artJ, astA, speA, adiA	FB Fibonacci
40	hisM, hisP, hisQ	FB Fibonacci
41	fdnI, fdhF, fdnH, fdnG, fdoI, fdoH, fdoG, focA, hycD, hycC, hycF, hycG, hycB, hycE, nrdD	FB Fibonacci
42	phoA, dxs, tpiA	FB Fibonacci
49	glyQ, glyS, gshB, ldtA, ldtB, ldtC, ldtD, ldtE, purD	Fibonacci
53	mnmG, mnmE	FB Fibonacci
54	alaB, alaA, alaC	FB Fibonacci
56	mhpF, aldB	FB Fibonacci
59	fau, pdxH	n = 0, l = 1
60	folD, metF, thyA	Fibonacci
63	talB, talA, tktA, tktB	FB Fibonacci
65	aroP, tnaB, trpS	Fibonacci
66	hxpA, ybiV, yidA, yigL	n = 0, l = 2
71	argI, argF	FB Fibonacci
72	potE, speC, speF	FB Fibonacci
73	hypF, pyrI, pyrB	FF Fibonacci
75	cadA, ldcC, cadB, lysO, lysP, lysU, lysS, tilS	FB Fibonacci
77	hisJ, hisS	FB Fibonacci
78	sucB, alkB, kgtP, menD, roxA, sucA, tauD	FB Fibonacci
79	argD, gabT, puuE, gdhA, gltD, gltB	FB Fibonacci
80	serC, arnB, hisC, patA, wecE	FB Fibonacci
83	ilvE, tyrB	FB Fibonacci
84	argA, gadA, gadB, gadC, glnA, gltS, gltX, gluQ, gshA, murI, proB, puuA, rimK	FB Fibonacci
85	gltL, gltJ, gltK, gltI, gltP	FB Fibonacci
90	relA, mnmC	FF Fibonacci
91	folA, folM	FB Fibonacci
92	mnaT, metG, metK, metQ, metI, metN, ybdL	FB Fibonacci
93	leuE, yjeH	FB Fibonacci
94	bisC, msrA, msrB, msrC	FB Fibonacci
95	metE, mmuM	FF Fibonacci
96	aphA, rnpA, rnpB, rnd, rnt, rph, rna	Fibonacci
97	queD, nudB	n = 0, l = 2
103	metA, rhtA, thrB, ygeA	Fibonacci
104	napA, napB, narI, narH, narG, narV, narZ, narY	Multi-Layer
105	narU, narK	n = 0, l = 1
106	ansB, ansA, iaaA, ansP, asnS	n = 1, l = 1

Table 4: List of building blocks for amino acid network

Block Id	Fiber Ids	Fiber Reg Ids	Fibered Nodes	Fiber Regs	Regulators	Class	r
0	14	60, 53	nudJ, spoT	folD, mnmE, mnmG	cycA, folC, gcvH, gcvP, gcvT, glpF, glyA, lpd, panB	FF Fibonacci	7.7631

Block Id	Fiber Ids	Fiber Reg Ids	Fibered Nodes	Fiber Regs	Regulators	Class	r
1	16	60, 53, 14	nrdA, nrdB, nrdE, nrdF	folD, mnmE, mnmG, spoT	cycA, folC, gcvH, gcvP, gcvT, glpF, glyA, lpd, panB, pnp	FF Fibonacci	7.7631
2	23, 56, 36, 78, 54, 79, 80, 83, 84, 85, 49	97	alaA, alaB, alaC, aldB, alkB, argA, argD, arnB, cysK, cysM, gabT, gadA, gadB, gadC, gdhA, glnA, gltB, gltD, gltI, gltJ, gltK, gltL, gltP, gltS, gltX, gluQ, glyQ, glyS, gshA, gshB, hisC, ilvE, kgtP, ldtA, ldtB, ldtC, ldtD, ldtE, malY, menD, metC, mhpF, murI, patA, proB, purD, puuA, puuE, rimK, roxA, serC, sucA, sucB, tauD, tyrB, wecE	queD	ackA, actP, adhE, argE, aspC, astC, astC, astD, astE, avtA, carA, carB, cycA, folE, folX, gcvP, glpF, glyA, glyA, metB, metH, poxB, purT, satP, sufS, tnaA, trpD, trpE	FB Fibonacci	25.9708
3	26, 54, 78, 84, 79, 80, 28, 85, 83, 49, 30, 37, 40, 71, 72, 75, 77		adiA, adiC, alaA, alaB, alaC, alkB, argA, argD, argF, argI, argS, arnB, artJ, artM, artP, artQ, astA, cadA, cadB, dauA, dctA, dcuA, dcuC, frdA, frdB, frdC, frdD, gabT, gadA, gadB, gadC, gdhA, glnA, gltB, gltD, gltI, gltJ, gltK, gltL, gltP, gltS, gltX, gluQ, glyQ, glyS, gshA, gshB, hisC, hisJ, hisM, hisP, hisQ, hisS, ilvE, kgtP, ldcC, ldtA, ldtB, ldtC, ldtD, ldtE, lysO, lysP, lysS, lysU, menD, murI, patA, potE, proB, purD, puuA, puuE, rimK, roxA, scpC, sdhA, sdhB, sdhC, sdhD, serC, speA, speC, speF, sucA, sucB, sucC, sucD, tauD, tilS, tyrB, wecE		argE, argH, argO, argT, aspC, astB, astC, astD, astE, avtA, carA, carB, citT, cycA, dcuB, epmB, gcvP, glpF, glyA, metB, nadB, satP, sufS, trpD, trpE, ttdT	FB Fibonacci	25.9721

Block Id	Fiber Ids	Fiber Reg Ids	Fibered Nodes	Fiber Regs	Regulators	Class	r
5	30, 54, 78, 84, 79, 80, 26, 28, 85, 83, 49		alaA, alaB, alaC, alkB, argA, argD, arnB, dauA, dctA, dcuA, dcuC, frdA, frdB, frdC, frdD, gabT, gadA, gadB, gadC, gdhA, glnA, gltB, gltD, gltI, gltJ, gltK, gltL, gltP, gltS, gltX, gluQ, glyQ, glyS, gshA, gshB, hisC, ilvE, kgtP, ldtA, ldtB, ldtC, ldtD, ldtE, menD, murI, patA, proB, purD, puuA, puuE, rimK, roxA, scpC, sdhA, sdhB, sdhC, sdhD, serC, sucA, sucB, sucC, sucD, tauD, tyrB, wecE		argH, aspC, astC, astD, astE, avtA, carA, carB, citT, cycA, dcuB, gcvP, glpF, glyA, metB, nadB, satP, sufS, trpD, trpE, ttdT	FB Fibonacci	25.9721
6	35		ilvB, ilvG, ilvH, ilvI, ilvM, ilvN, pflB, tdcE		avtA, metB, purT, satP	Fibonacci	2.4369
8	37, 71, 75, 77, 40, 72		adiA, adiC, argF, argI, argS, artJ, artM, artP, artQ, astA, cadA, cadB, hisJ, hisM, hisP, hisQ, hisS, ldcC, lysO, lysP, lysS, lysU, potE, speA, speC, speF, tilS		argE, argH, argO, argT, astB, astC, carA, carB, epmB	FB Fibonacci	12.3949
10	41, 35		fdhF, fdnG, fdnH, fdnI, fdoG, fdoH, fdoI, focA, hycB, hycC, hycD, hycE, hycF, hycG, ilvB, ilvG, ilvH, ilvI, ilvM, ilvN, nrdD, pflB, tdcE		avtA, folE, metB, purT, satP	FB Fibonacci	7.0095
11	42, 63		dxs, phoA, talA, talB, tktA, tktB, tpiA		gapA, trpA, trpB	FB Fibonacci	5.8481
12	49		glyQ, glyS, gshB, ldtA, ldtB, ldtC, ldtD, ldtE, purD		cycA, gcvP, glpF, glyA	Fibonacci	5.3957
13	53, 60, 91		folA, folD, folM, metF, mnmE, mnmG, thyA		cycA, folC, gcvH, gcvP, gcvT, glpF, glyA, lpd, panB	FB Fibonacci	8.3835

Block Id	Fiber Ids	Fiber Reg Ids	Fibered Nodes	Fiber Regs	Regulators	Class	r
14	54, 84, 79, 80, 85, 83, 78, 49		alaA, alaB, alaC, alkB, argA, argD, arnB, gabT, gadA, gadB, gadC, gdhA, glnA, gltB, gltD, gltI, gltJ, gltK, gltL, gltP, gltS, gltX, gluQ, glyQ, glyS, gshA, gshB, hisC, ilvE, kgtP, ldtA, ldtB, ldtC, ldtD, ldtE, menD, murI, patA, proB, purD, puuA, puuE, rimK, roxA, serC, sucA, sucB, tauD, tyrB, wecE		aspC, astC, astD, astE, avtA, carA, carB, cycA, gcvP, glpF, glyA, sufS, trpD, trpE	FB Fibonacci	25.9703
15	56, 78, 54, 79, 80, 83, 84, 85, 49	97	alaA, alaB, alaC, aldB, alkB, argA, argD, arnB, gabT, gadA, gadB, gadC, gdhA, glnA, gltB, gltD, gltI, gltJ, gltK, gltL, gltP, gltS, gltX, gluQ, glyQ, glyS, gshA, gshB, hisC, ilvE, kgtP, ldtA, ldtB, ldtC, ldtD, ldtE, menD, mhpF, murI, patA, proB, purD, puuA, puuE, rimK, roxA, serC, sucA, sucB, tauD, tyrB, wecE	queD	adhE, aspC, astC, astD, astE, avtA, avtA, carA, carB, cycA, folE, folX, gcvP, glpF, glyA, metB, poxB, satP, sufS, trpD, trpE	FB Fibonacci	25.9708
16	59		fau, pdxH		glyA	n = 0, l = 1	1
17	60		folD, metF, thyA		folC, gcvH, gcvP, gcvT, glyA, lpd, panB	Fibonacci	7.3549
19	65		aroP, tnaB, trpS		mtr, trpA, trpB	Fibonacci	4
20	66		hxpA, ybiV, yidA, yigL		trpA, trpB	n = 0, l = 2	2
23	73	37, 71, 75, 77, 40, 72	hypF, pyrB, pyrI	adiC, argF, argI, artJ, artM, artP, artQ, cadB, hisJ, hisM, hisP, hisQ, lysO, lysP, potE	argE, argH, argO, argT, carA, carB, epmB	FF Fibonacci	12.3946
32	90	60, 53	mnmC, relA	folD, mnmE, mnmG	cycA, folC, gcvH, gcvP, gcvT, glpF, glyA, lpd, panB	FF Fibonacci	7.7631
34	92, 94, 93	36, 95, 35	bisC, leuE, metG, metI, metK, metN, metQ, mnaT, msrA, msrB, msrC, ybdL, yjeH	malY, metC, metE, mmuM, pflB	avtA, metB, metH, miaB, moaA, phnJ, rimO, tnaA	FB Fibonacci	6.399
37	95	36	metE, mmuM	malY, metC	metB, tnaA	FF Fibonacci	3

Block Id	Fiber Ids	Fiber Reg Ids	Fibered Nodes	Fiber Regs	Regulators	Class	r
38	96		aphA, rna, rnd, rnpA, rnpB, rnt, rph		pnp	Fibonacci	1.618
39	97		nudB, queD		folE, folX	n = 0, l = 2	0
40	103		metA, rhtA, thrB, ygeA		metL, rhtB	Fibonacci	2.7321
41	104	105	napA, napB, narG, narH, narI, narV, narY, narZ	narK, narU	hmp	Multi-Layer	0
42	105		narK, narU		hmp	n = 0, l = 1	0
43	106		ansA, ansB, ansP, asnS, iaaA		asnA	n = 1, l = 1	1

Table 5: List of fibers in carbon network

Fiber Id	Nodes in Fiber	Class
15	bglF, crr, ptsG, glk, malQ, malX	FB Fibonacci
16	manX, manY, manZ	FB Fibonacci
22	otsB, treC	FB Fibonacci
23	fsaA, fsaB	FB Fibonacci
27	talB, talA	FB Fibonacci
28	tktA, tktB	FB Fibonacci
31	galK, galM	FB Fibonacci
32	mglB, mglC, mglA	FB Fibonacci
33	poxB, citC	FB Fibonacci
37	actP, satP	FB Fibonacci
40	idnK, gntK, gntU, gntT	FB Fibonacci
41	idnO, idnT	FB Fibonacci
44	dxs, tpiA	FB Fibonacci
48	araD, sgbE, ulaF, rpe	FB Fibonacci
49	edd, gnd, yieH	FB Fibonacci
54	aceB, glcB, gcl, ghrA, ghrB	FB Fibonacci
55	ybiV, yidA, yigL	n = 0, l = 9
61	setA, setB	Fibonacci
63	deoD, ppnP, nupG	n = 0, l = 1
64	nupC, rihC	FB Fibonacci
65	xapA, gsk, nepI	FB Fibonacci
66	rbsB, rbsC, rbsA	FF Fibonacci
67	glyA, folC, gcvT	Fibonacci
70	lpd, gcvP	FF Fibonacci
73	aldA, yqhD, yahK, ahr, ybbO	n = 0, l = 2
77	araA, araE, araF, araH, araG, ydeA	FB Fibonacci
79	fruA, fruB	n = 0, l = 1
80	araB, fucK	FB Fibonacci
81	alsB, alsC, alsA	n = 3, l = 0
84	ribB, rpiB, yajO	FB Fibonacci
85	lyxK, xylB	Multi-Layer
86	xylA, xylE, xylF, xylH, xylG	n = 5, l = 0

Table 6: List of building blocks for carbon network

Block Id	Fiber Ids	Fiber Reg Ids	Fibered Nodes	Fiber Regs	Regulators	Class	r
0	15, 22, 32, 31		bglF, crr, galK, galM, glk, malQ, malX, mglA, mglB, mglC, otsB, ptsG, treC		chbF, fucP, galP, gcd, lacY, lacZ, melA, melB, melB, otsA, otsA, pgi, phoA, treB, uhpT	FB Fibonacci	6.6985
1	16, 15, 22, 23, 27, 28, 44, 48, 32, 31, 40, 49, 41, 54, 84, 80, 77	85, 86	aceB, araA, araB, araD, araE, araF, araG, araH, bglF, crr, dxs, edd, fsaA, fsaB, fucK, galK, galM, gcl, ghrA, ghrB, glcB, glk, gnd, gntK, gntT, gntU, idnK, idnO, idnT, malQ, malX, manX, manY, manZ, mglA, mglB, mglC, otsB, ptsG, ribB, rpe, rpiB, sgbE, talA, talB, tktA, tktB, tpiA, treC, ulaF, yajO, ydeA, yieH	lyxK, xylA, xylB, xylE, xylF, xylG, xylH	alsE, chbF, eda, fucI, fucP, galP, gapA, gatY, gatZ, gcd, glmS, gntP, gutQ, idnD, kduD, lacY, lacZ, melA, melB, melB, mtlD, otsA, otsA, pgi, pgi, phoA, treB, uhpT	FB Fibonacci	10.9963
6	31, 32		galK, galM, mglA, mglB, mglC		fucP, galP, lacY, lacZ, melA, melB	FB Fibonacci	6.4283
8	33, 37		actP, citC, poxB, satP		ackA, chbG, nagA, purT	FB Fibonacci	3.9173
16	55		ybiV, yidA, yigL		eda, gatA, gatB, gatC, gatY, gatZ, srlA, srlB, srlE	n = 0, l = 9	0
17	61		setA, setB		lacY, lacZ	Fibonacci	3.7913
18	63		deoD, nupG, ppnP		add	n = 0, l = 1	0
19	64, 65		gsk, nepI, nupC, rihC, xapA		add	FB Fibonacci	NaN
21	66	64, 65	rbsA, rbsB, rbsC	nepI, nupC, rihC, xapA	add	FF Fibonacci	3
22	67		folC, gcvT, glyA		purH, purN	Fibonacci	3
23	70	67	gcvP, lpd	folC	purH, purN	FF Fibonacci	2
24	73		ahr, aldA, yahK, ybbO, yqhD		fucA, fucO	n = 0, l = 2	1.618
25	77, 32, 31		araA, araE, araF, araG, araH, galK, galM, mglA, mglB, mglC, ydeA		fucI, fucP, galP, lacY, lacZ, melA, melB, melB	FB Fibonacci	7.9554
26	79		fruA, fruB		lacZ	n = 0, l = 1	0
28	81		alsA, alsB, alsC			n = 3, l = 0	3
30	85	86	lyxK, xylB	xylA, xylE, xylF, xylG, xylH		Multi-Layer	5
31	86		xylA, xylE, xylF, xylG, xylH			n = 5, l = 0	5

Table 7: List of fibers in glycolysis network

Fiber Id	Nodes in Fiber	Class
1	gcd, galP	FB Fibonacci
2	bglF, crr, ptsG, manX, manY, manZ, glk, malQ, malX	FB Fibonacci
3	pgi, pfkB	FB Fibonacci
4	fsaA, fsaB	FB Fibonacci
6	talB, talA, tktA, tktB	FB Fibonacci
9	glcA, lldP	FB Fibonacci
10	glcF, glcE, glcD	FB Fibonacci
14	ppnP, xapA	FB Fibonacci
15	gsk, nepI	FB Fibonacci
17	cdd, rihA, rihB, udk	n = 0, l = 2
18	betA, emrE	FB Fibonacci
19	betT, yeaW, yeaX	FB Fibonacci
22	glpF, glpK	FB Fibonacci
23	eutC, eutB	n = 0, l = 2
24	cysJ, fre, ribF, hisD, nagE, cmtA, cmtB, garR, gatA, gatB, gatC, mak, mtlA, nupG, ompC, ompF, phoE, srlA, srlB, srlE	FB Fibonacci
25	dhaM, dhaL, dhaK	FF Fibonacci
26	pdxI, pdxK, pdxY	FF Fibonacci
29	cycA, pssA	FB Fibonacci
30	dsdA, dsdX, cysE, entB, entD, entF, entE, glyA, sdaB, sdaA, tdcG, sdaC, serS, sstT, trpB, ydfG	FB Fibonacci
31	tdcB, tdcC	FB Fibonacci
33	garK, glxK, glxR	FB Fibonacci
34	garP, gudP	FB Fibonacci
35	idnK, gntK, gntU, gntT	FB Fibonacci
36	idnO, idnT, kduD	FB Fibonacci
38	ilvA, ltaE, rhtC, tdh, yiaY, thrS	FB Fibonacci
39	rhtA, tsaC	FB Fibonacci
40	dxs, tpiA	FB Fibonacci
41	fbaA, fbaB	FB Fibonacci
43	fbp, glpX, ybhA, yggF	FB Fibonacci
44	kbaZ, kbaY, gatZ, gatY	FB Fibonacci
45	aroG, aroH, aroF	FF Fibonacci
48	gpmA, gpmM	FB Fibonacci
50	kdsA, murA, ppc, pykA, pykF	n = 0, l = 3
51	pgk, serA	FB Fibonacci
53	hxpA, ybiV, yidA, yigL	FB Fibonacci

Table 8: List of building blocks for glycolysis network

Block Id	Fiber Ids	Fiber Reg Ids	Fibered Nodes	Fiber Regs	Regulators	Class	r
0	1, 2, 6, 3, 4, 30, 24, 41, 40, 43, 44, 29, 31, 39, 38		bglF, cmtA, cmtB, crr, cycA, cysE, cysJ, dsdA, dsdX, dxs, entB, entD, entE, entF, fbaA, fbaB, fbp, fre, fsaA, fsaB, galP, garR, gatA, gatB, gatC, gatY, gatZ, gcd, glk, glpX, glyA, hisD, ilvA, kbaY, kbaZ, ltaE, mak, malQ, malX, manX, manY, manZ, mtlA, nagE, nupG, ompC, ompF, pfkB, pgi, phoE, pssA, ptsG, rhtA, rhtC, ribF, sdaA, sdaB, sdaC, serS, srlA, srlB, srlE, sstT, talA, talB, tdcB, tdcC, tdcG, tdh, thrS, tktA, tktB, tpiA, trpB, tsaC, ybhA, ydfG, yggF, yiaY		gapA, gldA, glmS, pfkA, phoA, trpA, uhpT	FB Fibonacci	13.7653
5	9, 10		glcA, glcD, glcE, glcF, lldP		actP, phoA	FB Fibonacci	5.2926
7	14, 15		gsk, nepI, ppnP, xapA		deoD, phoA	FB Fibonacci	2.3028
9	17		cdd, rihA, rihB, udk		nupC, phoA	n = 0, l = 2	0
10	18, 19		betA, betT, emrE, yeaW, yeaX		phoA	FB Fibonacci	2

Block Id	Fiber Ids	Fiber Reg Ids	Fibered Nodes	Fiber Regs	Regulators	Class	r
12	22, 53, 4, 41, 2, 3, 6, 30, 24, 40, 43, 44, 1, 29, 31, 39, 38		bglF, cmtA, cmtB, crr, cycA, cysE, cysJ, dsdA, dsdX, dxs, entB, entD, entE, entF, fbaA, fbaB, fbp, fre, fsaA, fsaB, galP, garR, gatA, gatB, gatC, gatY, gatZ, gcd, glk, glpF, glpK, glpX, glyA, hisD, hxpA, ilvA, kbaY, kbaZ, ltaE, mak, malQ, malX, manX, manY, manZ, mtlA, nagE, nupG, ompC, ompF, pfkB, pgi, phoE, pssA, ptsG, rhtA, rhtC, ribF, sdaA, sdaB, sdaC, serS, srlA, srlB, srlE, sstT, talA, talB, tdcB, tdcC, tdcG, tdh, thrS, tktA, tktB, tpiA, trpB, tsaC, ybhA, ybiV, ydfG, yggF, yiaY, yidA, yigL		clsB, gapA, gldA, glmS, pfkA, phoA, trpA, uhpT	FB Fibonacci	13.7691
13	23		eutB, eutC		clsB, phoA	n = 0, l = 2	0

Block Id	Fiber Ids	Fiber Reg Ids	Fibered Nodes	Fiber Regs	Regulators	Class	r
14	24, 33, 6, 3, 4, 34, 2, 30, 41, 40, 43, 44, 1, 29, 31, 39, 38		bglF, cmtA, cmtB, crr, cycA, cysE, cysJ, dsdA, dsdX, dxs, entB, entD, entE, entF, fbaA, fbaB, fbp, fre, fsaA, fsaB, galP, garK, garP, garR, gatA, gatB, gatC, gatY, gatZ, gcd, glk, glpX, glxK, glxR, glyA, gudP, hisD, ilvA, kbaY, kbaZ, ltaE, mak, malQ, malX, manX, manY, manZ, mtlA, nagE, nupG, ompC, ompF, pfkB, pgi, phoE, pssA, ptsG, rhtA, rhtC, ribF, sdaA, sdaB, sdaC, serS, srlA, srlB, srlE, sstT, talA, talB, tdcB, tdcC, tdcG, tdh, thrS, tktA, tktB, tpiA, trpB, tsaC, ybhA, ydfG, yggF, yiaY		gapA, gldA, glmS, pfkA, phoA, trpA, uhpT, uhpT	FB Fibonacci	13.7793
15	25	41, 43, 4, 1, 44, 24, 2, 3, 6, 40, 30, 29, 31, 39, 38	dhaK, dhaL, dhaM	cycA, dsdX, fbaA, fbaB, fbp, fsaA, fsaB, galP, gatY, gatZ, gcd, glpX, glyA, kbaY, kbaZ, mak, malQ, malX, manX, manY, manZ, pfkB, pgi, pssA, rhtA, rhtC, sdaC, sstT, talA, talB, tdcC, tktA, tktB, tpiA, trpB, tsaC, ybhA, yggF	aroA, eno, gapA, gldA, glmS, pfkA, phoA, ppsA, trpA, uhpT	FF Fibonacci	13.7653
16	26	43, 53, 3, 41, 2, 4, 6, 24, 30, 40, 44, 1, 29, 31, 39, 38	pdxI, pdxK, pdxY	cycA, dsdX, fbaA, fbaB, fbp, fsaA, fsaB, galP, gatY, gatZ, gcd, glpX, glyA, kbaY, kbaZ, mak, malQ, malX, manX, manY, manZ, pfkB, pgi, pssA, rhtA, rhtC, sdaC, sstT, talA, talB, tdcC, tktA, tktB, tpiA, trpB, tsaC, ybhA, yggF, yigL	gapA, gldA, glmS, pfkA, phoA, trpA, uhpT	FF Fibonacci	13.7653

Block Id	Fiber Ids	Fiber Reg Ids	Fibered Nodes	Fiber Regs	Regulators	Class	r
17	29, 30, 31, 39, 38		cycA, cysE, dsdA, dsdX, entB, entD, entE, entF, glyA, ilvA, ltaE, pssA, rhtA, rhtC, sdaA, sdaB, sdaC, serS, sstT, tdcB, tdcC, tdcG, tdh, thrS, trpB, tsaC, ydfG, yiaY		phoA, trpA	FB Fibonacci	7.2031
20	33, 34, 24		cmtA, cmtB, cysJ, fre, garK, garP, garR, gatA, gatB, gatC, glxK, glxR, gudP, hisD, mak, mtlA, nagE, nupG, ompC, ompF, phoE, ribF, srlA, srlB, srlE		phoA	FB Fibonacci	3.0739
22	35, 36		gntK, gntT, gntU, idnK, idnO, idnT, kduD		gntP, phoA	FB Fibonacci	4.6458
30	45	41, 43, 4, 1, 44, 24, 2, 3, 6, 40, 30, 29, 31, 39, 38	aroF, aroG, aroH	cycA, dsdX, fbaA, fbaB, fbp, fsaA, fsaB, galP, gatY, gatZ, gcd, glpX, glyA, kbaY, kbaZ, mak, malQ, malX, manX, manY, manZ, pfkB, pgi, pssA, rhtA, rhtC, sdaC, sstT, talA, talB, tdcC, tktA, tktB, tpiA, trpB, tsaC, ybhA, yggF	aroA, eno, gapA, gldA, glmS, pfkA, phoA, ppsA, trpA, uhpT	FF Fibonacci	13.7653
31	48, 33, 34, 24, 51		cmtA, cmtB, cysJ, fre, garK, garP, garR, gatA, gatB, gatC, glxK, glxR, gpmA, gpmM, gudP, hisD, mak, mtlA, nagE, nupG, ompC, ompF, pgk, phoE, ribF, serA, srlA, srlB, srlE		eno, phoA	FB Fibonacci	5
32	50		kdsA, murA, ppc, pykA, pykF		aroA, eno, ppsA	n = 0, l = 3	1.618

Block Id	Fiber Ids	Fiber Reg Ids	Fibered Nodes	Fiber Regs	Regulators	Class	r
34	53, 41, 43, 4, 1, 44, 24, 2, 3, 6, 40, 30, 29, 31, 39, 38		bglF, cmtA, cmtB, crr, cycA, cysE, cysJ, dsdA, dsdX, dxs, entB, entD, entE, entF, fbaA, fbaB, fbp, fre, fsaA, fsaB, galP, garR, gatA, gatB, gatC, gatY, gatZ, gcd, glk, glpX, glyA, hisD, hxpA, ilvA, kbaY, kbaZ, ltaE, mak, malQ, malX, manX, manY, manZ, mtlA, nagE, nupG, ompC, ompF, pfkB, pgi, phoE, pssA, ptsG, rhtA, rhtC, ribF, sdaA, sdaB, sdaC, serS, srlA, srlB, srlE, sstT, talA, talB, tdcB, tdcC, tdcG, tdh, thrS, tktA, tktB, tpiA, trpB, tsaC, ybhA, ybiV, ydfG, yggF, yiaY, yidA, yigL		gapA, gldA, glmS, pfkA, phoA, trpA, uhpT	FB Fibonacci	13.7668

Table 9: List of fibers in oxidative network

Fiber Id	Nodes in Fiber	Class
7	acnB, acnA, aceA, icd	n = 4, l = 0
8	poxB, citC	FB Fibonacci
9	cyoC, cyoB, cyoA, cyoD, cydX, cydB, cydA, appB, appC, napH, napG, narI, narH, narG	FF Fibonacci
13	actP, satP	FB Fibonacci
16	dcyD, tnaA, yhaM, cysS, dfp	FF Fibonacci
17	metC, malY, aspC, gshA, metB	FB Fibonacci
18	cydC, cydD	FB Fibonacci
19	iscS, sufS	FF Fibonacci
20	tcyJ, tcyL, tcyN	FB Fibonacci
22	frdD, frdC, frdB, frdA, dcuC, fumC	FB Fibonacci
23	dauA, dctA, dcuA, dcuB, nadB	FB Fibonacci
25	ccp, ftnA, adeD, katE, katG	FF Fibonacci
26	fdnI, fdhF, fdnH, fdnG, fdoI, fdoH, fdoG, focA, hycD, hycC, hycF, hycG, hycB, hycE, nrdD	n = 7, l = 1
28	fsaA, fsaB	Fibonacci
30	pfkA, pfkB	FF Fibonacci
33	idnK, gntK, gntU, gntT	FB Fibonacci
34	idnO, idnT	FB Fibonacci
39	kdgK, kdgT	Fibonacci
41	eamA, eamB	FB Fibonacci
42	chaC, ggt, gloA, grxA, gsiC, gsiD, gsiB, gsiA, gstA, yfcF, gstB, yfcG, yghU, yqjG	FB Fibonacci
45	bioB, miaB, rimO	FF Fibonacci
46	alaB, alaA, alaC, alaE, alaS, alr, dadX, bioF, murC	FF Fibonacci

Fiber Id	Nodes in Fiber	Class
48	cysI, cysJ	FF Fibonacci
49	selD, lipA, ttcA, tusA, ynjE	FF Fibonacci
50	ydbK, ispG, nrdG, pflA	Fibonacci
52	acrB, acrA, entS, fes	Fibonacci
54	macA, macB	Fibonacci
55	ompC, ompF, phoE	Fibonacci
59	aphA, umpG, umpH, ushA, yjjG	n = 0, l = 1
60	mntH, mntP	n = 2, l = 0

Table 10: List of building blocks for oxidative network

Block Id	Fiber Ids	Fiber Reg Ids	Fibered Nodes	Fiber Regs	Regulators	Class	r
0	7		aceA, acnA, acnB, icd			n = 4, l = 0	4
1	8, 13		actP, citC, poxB, satP		ackA, cysK, cysM, purT	FB Fibonacci	5.6543
2	9	8, 13	appB, appC, cydA, cydB, cydX, cyoA, cyoB, cyoC, cyoD, napG, napH, narG, narH, narI	actP, poxB, satP	ackA, cysK, cysM, purT	FF Fibonacci	5.6543
4	16	17, 18, 41, 42, 20	cysS, dcyD, dfp, tnaA, yhaM	aspC, cydC, cydD, eamA, eamB, grxA, gsiA, gsiB, gsiC, gsiD, metB, tcyJ, tcyL, tcyN	cysK, cysM, gor, gss, tcyP	FF Fibonacci	10.3686
5	17, 18, 41, 42, 20		aspC, chaC, cydC, cydD, eamA, eamB, ggt, gloA, grxA, gshA, gsiA, gsiB, gsiC, gsiD, gstA, gstB, malY, metB, metC, tcyJ, tcyL, tcyN, yfcF, yfcG, yghU, yqjG		cysK, cysM, gor, gss, tcyP	FB Fibonacci	10.3686
7	19	17, 18, 41, 42, 20	iscS, sufS	aspC, cydC, cydD, eamA, eamB, grxA, gsiA, gsiB, gsiC, gsiD, metB, tcyJ, tcyL, tcyN	cysK, cysM, gor, gss, tcyP	FF Fibonacci	10.3686
9	22, 23		dauA, dctA, dcuA, dcuB, dcuC, frdA, frdB, frdC, frdD, fumC, nadB			FB Fibonacci	8.7446
11	25	23, 22	adeD, ccp, ftnA, katE, katG	dauA, dctA, dcuA, dcuB, dcuC, frdA, frdB, frdC, frdD, fumC, nadB	sodA	FF Fibonacci	3
12	26		fdhF, fdnG, fdnH, fdnI, fdoG, fdoH, fdoI, focA, hycB, hycC, hycD, hycE, hycF, hycG, nrdD		ribA	n = 7, l = 1	7
13	28		fsaA, fsaB		glmS, pgi, phoA	Fibonacci	3.3176
14	30	28	pfkA, pfkB	fsaA, fsaB	glmS, pgi, phoA, uhpT	FF Fibonacci	3.7172

Block Id	Fiber Ids	Fiber Reg Ids	Fibered Nodes	Fiber Regs	Regulators	Class	r
15	33, 34		gntK, gntT, gntU, idnK, idnO, idnT		gntP, kduD, phoA	FB Fibonacci	4.8541
17	39		kdgK, kdgT		kduD, uxuA, yagE	Fibonacci	2.4142
20	45	17, 18, 41, 42, 19, 20	bioB, miaB, rimO	aspC, cydC, cydD, eamA, eamB, grxA, gsiA, gsiB, gsiC, gsiD, iscS, metB, sufS, tcyJ, tcyL, tcyN	cysK, cysM, gor, gss, tcyP	FF Fibonacci	10.3686
21	46	17, 18, 41, 42, 19, 20	alaA, alaB, alaC, alaE, alaS, alr, bioF, dadX, murC	aspC, cydC, cydD, eamA, eamB, grxA, gsiA, gsiB, gsiC, gsiD, iscS, metB, sufS, tcyJ, tcyL, tcyN	cycA, cysK, cysM, gor, gss, tcyP	FF Fibonacci	10.3686
22	48	42, 19, 18, 17, 20, 41	cysI, cysJ	aspC, cydC, cydD, eamA, eamB, grxA, gsiA, gsiB, gsiC, gsiD, metB, sufS, tcyJ, tcyL, tcyN	cysK, cysM, gor, gss, tcyP	FF Fibonacci	10.3686
23	49	19, 17, 18, 20, 41, 42	lipA, selD, ttcA, tusA, ynjE	aspC, cydC, cydD, eamA, eamB, grxA, gsiA, gsiB, gsiC, gsiD, metB, sufS, tcyJ, tcyL, tcyN	cysK, cysM, gor, gss, tcyP	FF Fibonacci	10.3686
24	50		ispG, nrdG, pflA, ydbK		fpr	Fibonacci	1.618
25	52		acrA, acrB, entS, fes		tolC	Fibonacci	4
26	54		macA, macB		tolC	Fibonacci	3
27	55		ompC, ompF, phoE		ompN	Fibonacci	2.3028
28	59		aphA, umpG, umpH, ushA, yjjG		waaZ	$n = 0, l = 1$	0
29	60		mntH, mntP			$n = 2, l = 0$	2
